# A feedback loop of conditionally stable circuits drives the cell cycle from checkpoint to checkpoint

**DOI:** 10.1038/s41598-019-52725-1

**Published:** 2019-11-11

**Authors:** Dávid Deritei, Jordan Rozum, Erzsébet Ravasz Regan, Réka Albert

**Affiliations:** 1 Department of Physics, Pennsylvania State University, University Park, PA, United States of America; 2 Department of Network and Data Science, Central European University, Budapest, Hungary; 3 Biochemistry and Molecular Biology, The College of Wooster, Wooster, OH United States of America

**Keywords:** Regulatory networks, Oscillators, Modularity, Computer modelling, Logic gates

## Abstract

We perform logic-based network analysis on a model of the mammalian cell cycle. This model is composed of a Restriction Switch driving cell cycle commitment and a Phase Switch driving mitotic entry and exit. By generalizing the concept of stable motif, i.e., a self-sustaining positive feedback loop that maintains an associated state, we introduce the concept of a conditionally stable motif, the stability of which is contingent on external conditions. We show that the stable motifs of the Phase Switch are contingent on the state of three nodes through which it receives input from the rest of the network. Biologically, these conditions correspond to cell cycle checkpoints. Holding these nodes locked (akin to a checkpoint-free cell) transforms the Phase Switch into an autonomous oscillator that robustly toggles through the cell cycle phases G1, G2 and mitosis. The conditionally stable motifs of the Phase Switch Oscillator are organized into an ordered sequence, such that they serially stabilize each other but also cause their own destabilization. Along the way they channel the dynamics of the module onto a narrow path in state space, lending robustness to the oscillation. Self-destabilizing conditionally stable motifs suggest a general negative feedback mechanism leading to sustained oscillations.

## Introduction

Bottom-up mechanistic models of biological regulation are becoming increasingly adept at describing and predicting complex cellular behavior^1–5^. For example, mechanistic models of the control circuitry of the cell division cycle revealed that cells are making binary decisions whether to progress in the cycle^6–8^. As qualitative experimental data on regulatory interactions are more abundant than kinetic information, modeling frameworks that leverage qualitative data are often used to probe the behavior of large regulatory networks. Boolean models, in particular, assume that the essential logic by which regulatory interactions drive cell behavior can be adequately described by a network of molecular components (e.g. mRNA, transcription factors, signaling proteins), where the activity of each is described by a binary variable. Boolean models have been shown to faithfully reproduce cellular phenotypes and behaviors in cells from all domains of life^9–19^. For example, a 21-node Boolean model of the mammalian cell cycle reproduced the continuous cycling of cells in the presence of growth factors and their quiescent state when the environment lacks growth factors^17^. Overall, the successes of Boolean modeling reveal that complex, context-dependent cellular behaviors generally do not rely on narrowly tuned kinetic parameters. Rather, the network topology and combinatorial logic of regulatory interactions are key determinants of cellular function.

The idea that the structure of a regulatory circuit is key to its dynamic behavior inspired a series of methods to link features of network topology to biological insight^20–30^. An especially important, yet also challenging goal is to identify the repertoire of cellular phenotypes (e.g. cell cycle progression, cell cycle arrest, programmed cell death) and the decisions or trajectories that lead to each phenotype. Dynamic models represent such phenotypes as *attractors* (e.g. steady states or sustained oscillations). Identification and interpretation of model trajectories, and of stochastic oscillations in particular, is challenging even for Boolean models, because the state space grows exponentially with the number of modeled components. A recently introduced method to efficiently determine the attractor (phenotype) repertoire of a Boolean model of an interacting system of molecules relies on the construction of an *expanded network* that integrates the interaction network and the regulatory logic^31^. Analysis of the expanded network can identify subsystems called *stable motifs* that stabilize independently of the rest of the system. The sequential lock-in of stable motifs restricts the system’s dynamics until it reaches an attractor. Analysis of the expanded network of various signal transduction networks identified the stable motifs that determine the transition between epithelial and mesenchymal cell types^16^, the closure of stomatal pores in response to external or internal signals^18^, and the choice between apoptosis and survival of T cells^32^. A counterpart to stable motifs, *oscillating motifs*, are node subsets that cannot achieve a steady state, and thus give rise to oscillating or complex attractors^31,33^.

A benefit of logic-based structural analysis is that it can reveal the dynamical building blocks, or *modules*, that cellular regulation uses to drive distinct outcomes. Indeed, multiple lines of evidence suggest that cell-wide interaction networks are made of small tightly connected modules, linked into a hierarchy of looser modules at each scale^34–38^. Cells respond to their environment with discrete combinations of specific functions and often break down one functional module at a time^39,40^. Deritei *et al*. argued in 2016 that cellular regulatory networks exhibit *dynamical modularity*^17^: they are composed of a hierarchy of coupled modules, each of which is responsible for a discrete set of mutually exclusive phenotypic outcomes, such as survival vs. apoptosis (programmed cell death), cell cycle progression vs. cell cycle arrest. The global fate of cells then consists of distinct combinations of module states.

Dynamical modularity also explains biological rhythms generated by coupled modules. Indeed, the mammalian cell cycle model of Deritei *et al*. contains two main modules, each of which has multiple steady states. The two modular switches–the *Restriction Switch* responsible for cell cycle commitment and the *Phase Switch* in control of entry and exit from mitosis (cell division)–toggle each other such that the cell state follows a repeating sequence of distinct combinations of the switches’ steady states. Moreover, a significantly larger five-module Boolean model of cell cycle coordination with growth factor signaling shows similarly modular dynamics^19^.

Here we perform logic-based structural analysis of the cell cycle model of Deritei *et al*. to identify the stable motifs that drive switch states and to pinpoint the oscillating motif responsible for the cell cycle. A possible mechanism for a dynamically modular cycle is the existence of long, cross-module negative feedback loops in which a steady state of one switch toggles the state of a second switch, which then destabilizes the first. While inter-module feedback is indeed present, here we show that the Phase Switch *alone* has internal structural features capable of generating a robust cell cycle oscillation. We characterize these structural features by introducing the concept of conditionally stable motif, and by a coarse-graining that naturally emerges from the system’s regulatory logic.

### Background on the cell cycle model of Deritei *et al*

The cell division cycle is the series of events that yields the duplication of the cell’s DNA and division of its cytoplasm and organelles to produce two daughter cells. The eukaryotic cell cycle consists of five distinct phases: the first growth phase (G1); the DNA synthesis (S) phase; the G2 phase, during which the cells are growing rapidly and the microtubules begin to reorganize; mitosis, when the chromosomes separate and are pulled apart by microtubules; and cytokinesis, when all cell components separate. Activation of each phase depends on the proper completion of the previous phase, and this progression is monitored through three main checkpoints: the restriction point prior to DNA synthesis, the DNA damage checkpoint prior to mitosis and the spindle assembly checkpoint during mitosis. Cells that stop dividing enter a quiescent (G0) state.

**Figure 1:**
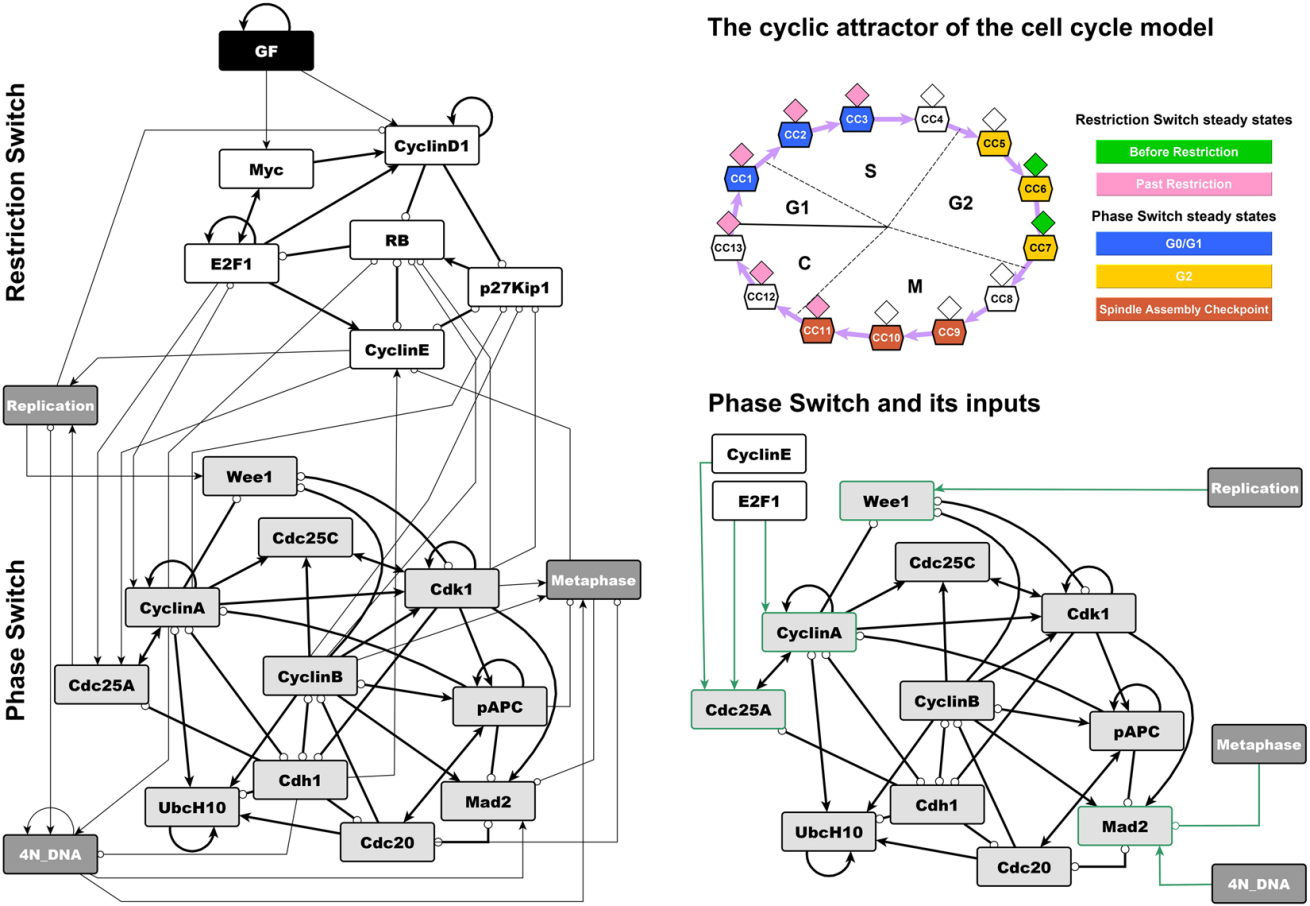
Illustration of the cell cycle model of Deritei *et al*.^17^ and its modules. The network contains modules that underlie two switches, the Restriction Switch (shown with white node background) and the Phase Switch (nodes with light grey background). The network also contains a Growth Factor input node (black), and three abstract nodes (dark grey) that stand for cell cycle processes (Replication, Metaphase) or global cell states (4N_DNA) that are triggered, monitored and terminated by the two controller switches. The bottom right panel shows the Phase Switch module and its inputs from the rest of the network (highlighted with green edges). All the inputs of the Phase Switch affect only four nodes: Cdc25A, CyclinA, Wee1 and Mad2 (green outline). Edges with terminal arrows indicate positive regulation, edges that end in open circles indicate negative regulation, and edges that have endings on both sides (i.e., bidirectional edges) indicate the superposition of two edges with opposite directions. The top right panel illustrates that in the presence of growth factors the model’s states form a cycle that progresses through the phases of the cell cycle, while toggling the steady states of the two modules. Each symbol represents a model state and separately encodes the state of the Restriction Switch (diamond) and the Phase Switch (hexagon). The color of each shape indicates that the corresponding state is close to one of the steady states indicated in the legend. For details please see Supplementary Note S1, and Fig. 2 of Deritei *et al*.^17^.

The Boolean cell cycle model by Deritei *et al*.^17^ builds on previously published models^6–8,12,41,42^ to synthesize the regulatory logic by which the core mammalian cell cycle control machinery generates the coordinated oscillatory activity patterns required to drive as well as monitor healthy cell cycle progression (Fig. 1, left). This model leverages the switch-like nature of checkpoint passages from G1 to S^43,44^, from G2 to mitosis^45,46^, as well as through the spindle assembly checkpoint^47^ to group the molecules controlling cell cycle progression into two semi-independent but coupled switches: the Restriction Switch and the Phase Switch (Fig. 1). The Restriction Switch (the six white nodes in Fig. 1) contains the molecular pathways leading to restriction point passage upon growth stimulation, and it has two steady states that correspond to cell states prior to, and following, commitment to cell cycle progression irrespective of ongoing growth stimulation. The Phase Switch (the 11 grey nodes in Fig. 1) is a synthesis of the positive feedback mechanisms among mitotic cyclin/cyclin-dependent kinase complexes. This module has three steady states corresponding to the state of cells arrested at one of three checkpoints (Supplementary Table S1): in G2 before the DNA damage checkpoint is cleared, at the spindle assembly checkpoint, and in G1. When the two switches are coupled via direct interactions as well as via three nodes that track the process of DNA replication and spindle assembly, they toggle each other in a sequence that mimics the cell cycle (see top right panel of Fig. 1). Supplementary Note S1 presents the details of the model, including a discussion of the Restriction Switch, Phase Switch and the implementation of the cell cycle checkpoints, as well as the sequence of states shown in the top right panel of Fig. 1.

### Key concepts in Boolean modeling

Boolean models of biological regulatory systems have been described in several recent review articles^48–51^. Here we review key concepts of Boolean modeling (see also the glossary of Supplementary Note S10 for a summary of the concepts discussed in this section). Boolean models assume that the activity of each element can be described by two qualitative states: 1 (interpreted as on, or active) and 0 (interpreted as off, or inactive). The future state of each node depends on the current state of its regulators (parents in the network) in a way described by a *regulatory function* ${x}^{\ast }={f}_{x}(Par(x))$, where for simplicity the state of the node is denoted with the node name, x^*^ is the next state of node $x$, *f*_*x*_ is the regulatory function of *x* and *Par*(*x*) represents the parent nodes of (nodes with edges pointing toward) *x*. The state of a Boolean system is defined as the vector formed by the on/off state of all the nodes. The order in which the nodes of a Boolean model are updated can significantly impact the emergent dynamical trajectories. The Deritei *et al*. model^17^ of the cell cycle used *synchronous update*, in which all nodes are updated at the same time and their next state is determined by the previous state of the system. Synchronous update yields deterministic trajectories. In this paper we use the *general asynchronous update* scheme, where the next node to be updated is chosen randomly, which adds a large degree of stochasticity into the emergent trajectory of the model. By comparing the results of synchronous and asynchronous update, we can determine the robustness of the trajectories to changes in the timing of individual events.

All trajectories of Boolean systems ultimately lead into attractors. An attractor is a single state, or a repeating set of states, that the system cannot leave. Attractors that are single states are called point attractors (or steady states). Point attractors of Boolean models of biological systems represent stable phenotypes. Attractors that contain multiple states are called complex attractors (or limit cycles in case of synchronous update). Limit cycles usually correspond to oscillations such as the cell cycle. The *state transition graph (STG)* is the network representing all the possible transitions between the states of a system. Point attractors correspond to sink nodes (i.e. nodes lacking outgoing edges) of the STG. Complex attractors correspond to terminal strongly connected components of the STG (i.e., there are no state transitions that lead out of the attractor). The point attractors of Boolean models are independent of update scheme, but the complex attractors may change or disappear when using different types of update.

**Figure 2:**
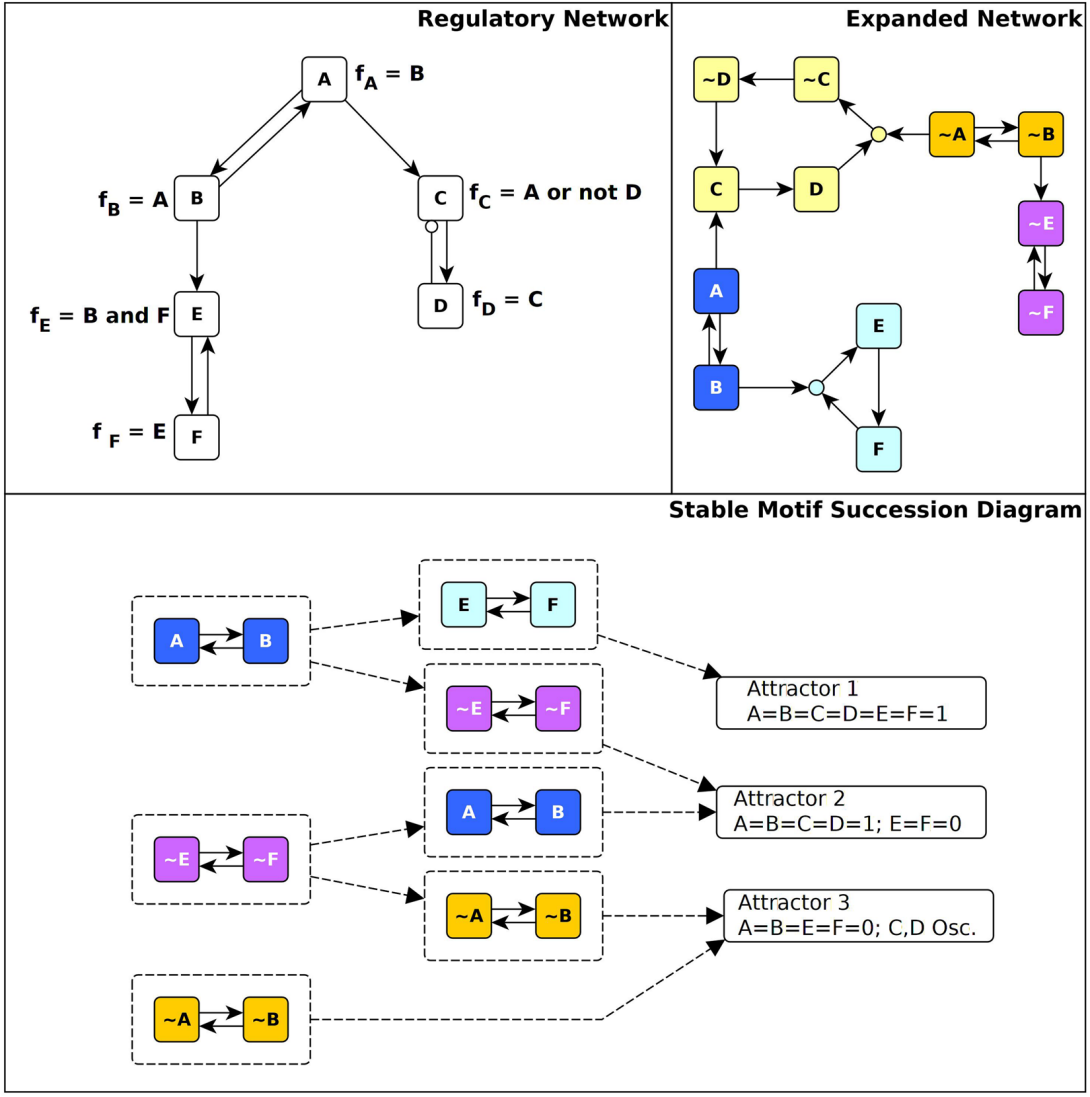
Illustration of the expanded network, stable motifs and conditionally stable motifs of a hypothetical network. In the network (shown on the top left panel) edges with terminal arrows indicate positive regulation and edges that end in open circles indicate negative regulation. The regulatory function of each node is indicated next to the node. The expanded network (top right panel) includes two virtual nodes for each node: the virtual node marked by the node name indicates the on (1) state of the node while the virtual node marked by the node name preceded by ~indicates the off (0) state of the node. The expanded network represents each AND gate with a composite node (small filled circle), e.g. the composite node with light blue background indicates the AND gate in the regulatory function of node E. (Recall that an OR gate in a Boolean rule corresponds to an AND gate in its negation, as seen in the case of the other, yellow composite node). The positive feedback loop between A and B yields two stable motifs, one corresponding to the on state of both nodes (shown in blue) and one corresponding to the off state of both nodes (shown in orange). The positive feedback between E and F can sustain their off state (stable motif shown in purple) but the on state of E and F can only be sustained if B is on. Thus, the virtual nodes E and F form a conditionally stable motif conditioned on B (light blue). The negative feedback between C and D leads to sustained oscillation of these two nodes (indicated by the cycle in yellow) if A = 0. If A = 1 C will also converge to 1 (see the edge from A to C in the expanded network). The stable motif succession diagram shown in the bottom panel indicates the possible sequences of successive stabilization of the three stable motifs and of the conditionally stable motif as well as the resulting attractor repertoire of the system.

Much of our analyses rely on the construction and properties of an *expanded network*^9,31,33,52,53^. The expanded network of a Boolean system encodes the causal relationships between node states reflected in the regulatory functions. (In technical terms, the expanded network is a one-to-one mapping of the regulatory functions written in a Blake canonical form^54^). The expanded network consists of two “virtual nodes” for each node (one for each of the two possible states) and “composite nodes” that embody AND gates among two or more node states. An edge from a virtual node to a composite node indicates that the virtual node is a necessary condition for states described by the composite node. An edge from any node to a virtual node indicates that the parent node is a sufficient condition for the state represented by the child node. Figure 2 indicates the expanded network that corresponds to the network and regulatory functions of a hypothetical example.

Within an expanded network, there are subgraphs that capture important dynamical features of the underlying system. Of particular interest here is the class of subgraphs called *stable motifs*^31^. A stable motif is a nonempty subgraph of an expanded network that satisfies four properties: (1) it is *strongly connected* (there is a path between every pair of nodes in the subgraph), (2) it is *consistent* (all represented conditions can be simultaneously satisfied), (3) it is *composite-closed* (if a composite node is in the subgraph, so too are all its virtual node parents), and (4) it is *minimal* (it contains no nontrivial subgraphs satisfying the first three properties). A stable motif determines a (non-empty) region of the state space (i.e., a trap space) from which dynamical trajectories cannot escape. In general, this region constrains some of the system variables (including the variables of the stable motif), but not others. The confinement of the constrained variables will be independent of the unconstrained variables even when these unconstrained variables are externally manipulated.

Another important class of expanded network subgraph is the oscillating motif. Like stable motifs, oscillating motifs are strongly connected, composite-closed subgraphs. Unlike stable motifs, however, oscillating motifs violate the consistency criterion in that every virtual node in the subgraph has its negation in the subgraph as well. Furthermore, an oscillating motif may not contain any stable motifs as subgraphs. An oscillating motif is so-named because its presence implies the existence of a complex attractor in which all of the nodes represented in the motif oscillate^31^.

The effects of the system entering a stable motif trap space can be evaluated by substituting the node states of a stable motif into the Boolean regulatory functions of the rest of the system and simplifying the regulatory functions using Boolean logic. These reduced functions describe the system’s evolution sufficiently long after the trap space has been entered. Using these rules, a new expanded network can be generated, in which additional stable motifs may be identified. The succession of stable motif identification and network reduction ends when there are no longer any stable motifs (when this occurs, the expanded network is either empty or it likely is an oscillating motif^31^). At each step in the succession, there may be several stable motifs; by considering all possible orders of stable motif selection, the attractors of the Boolean system can be enumerated. The bottom panel of Fig. 2 illustrates this procedure and the resulting paths to the system’s three attractors.

In this work, we introduce a new generalization of stable motifs that allows node states to stabilize when a specified set of conditions are satisfied. In particular, we define “conditionally stable motifs” (CSMs) to be consistent, strongly connected components of the expanded network that are not composite closed. For a given CSM, we define the CSM conditions to be the set of virtual nodes that are external to the CSM but are parents of composite nodes internal to the CSM. A stable motif can be viewed as a CSM with an empty condition set. A CSM becomes a stable motif under the assumption that its conditions are satisfied. We provide a method to construct all CSMs in a Boolean network and implement this method on the Phase Switch Oscillator (see Methods). The number of CSMs can become very large, which complicates interpretation of the collective significance of a system’s CSMs. For this reason, we focus our attention on two classes of CSM: those with the fewest conditions and those that are as large as possible.

### Three stable motifs and four conditionally stable motifs determine the three point attractors of the Phase Switch

**Figure 3:**
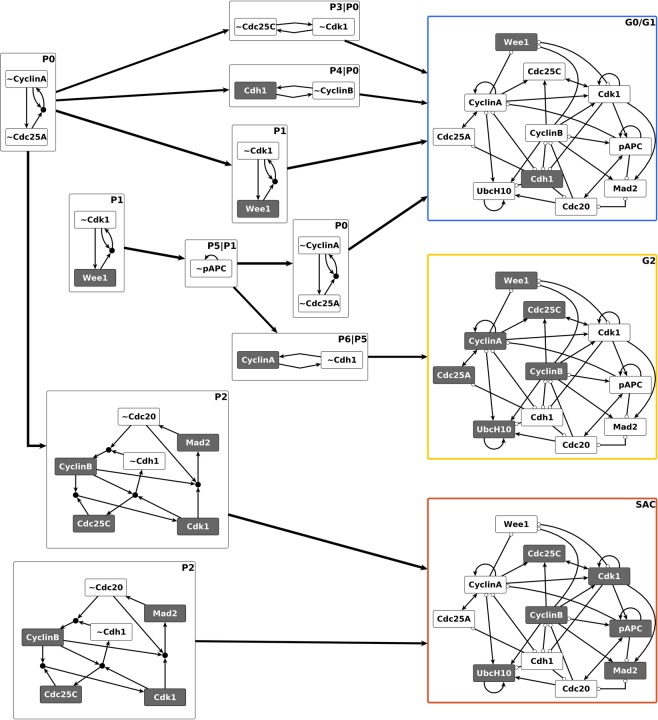
Distinct sequences of stable and conditionally stable motifs commit the Phase Switch to its attractor states. The stable motifs are shown in the expanded network formalism where a virtual node labeled by the name of the corresponding node represents the state 1 of the node (dark grey background); a virtual node labeled by the node name preceded by ~represents the state 0 of the node (white background). Stable motifs P0, P1 and P2 are the stable motifs of the Phase Switch when considered in isolation. Each path in the diagram begins at one of these stable motifs, and (conditionally) stable motifs in the path are self-sustaining when those earlier in the path are locked in. Each path terminates in one of the three point attractors (G0/G1, G2, or SAC), visualized in the Phase Switch regulatory network using white background for the off state of a node and dark grey for the on state. The outline color of the three attractors corresponds to the color-code used for differentiating the three attractors in Deritei *et al*.^17^.

In order to understand the structural causes of the toggle between the two modules of the cell cycle model, we first focused on characterizing the Phase Switch, i.e. the network among the 11 nodes with light grey background on Fig. 1. For this analysis we follow Deritei *et al*. in severing the inputs to the Phase Switch (see Supplementary Note S2 for a discussion of the assumptions behind this). The interactions among the 11 nodes of the Phase Switch express either positive or negative regulatory effects mediated by protein-protein binding, complex formation, and post-translational modifications (see Fig. 1 and Supplementary Note S1). The Phase Switch is strongly connected; all nodes within the module can be reached from all other nodes via at least one directed path. It contains both positive and negative feedback loops of various lengths. We used the expanded network formalism to express the regulatory functions of the Phase Switch (given in Supplementary Note S3); Supplementary Fig. S1 depicts the resulting expanded network. We identified three stable motifs, P0 to P2, as shown on Fig. 3. Additionally, there are four conditionally stable motifs: P3, P4, P5 and P6. Conditionally stable motifs P3 and P4 depend on the prior establishment of CyclinA = 0 (which is part of the P0 motif). We represent this dependence on the prior locking in of the P0 motif as “P3|P0” and “P4|P0”, respectively, in the motif label. Conditionally stable motif P5 is a stable motif only if P1 is already established (denoted P5|P1). Conditionally stable motif P6 is a stable motif only if P1 and P5 are already established (denoted P6|P5).

The stable motif succession diagram of the Phase Switch (Fig. 3) illustrates the relationship between stable motifs, conditionally stable motifs, and the three point attractors previously identified by Deritei *et al*. Each of these attractors represent cells arrested before one of three checkpoints: the restriction point in G1, the DNA damage checkpoint in G2, and the spindle assembly checkpoint (SAC), see Supplementary Table S1. Each stable motif in the diagram represents a commitment in the dynamics of the switch. For instance, because in the diagram there is no path from P0 (i.e., the inactivation of Cdc25A and CyclinA) to the G2 state, wherein CyclinA is active, the G2 state is not attainable when P0 has been locked in. Similarly, the SAC state is not attainable when P1 is active. In the SAC state CyclinA is inactive, the CyclinB/Cdk1 complex is active, and most importantly Mad2 is active, indicating the existence of unattached kinetochores on the cell’s replicated chromosomes. The succession diagram indicates that only the SAC state is available when the P2 motif, which includes active Mad2, is locked in, consistent with the spindle checkpoint role of Mad2. Figure 3 thus identifies the attractors of the Phase Switch with combinations of stable motifs and conditionally stable motifs. The G0/G1 attractor of the Phase Switch is reached by multiple trajectories for which the P0 motif stabilizes together with P1, P3, or P4. Alternatively, stabilization of P0 could be paired with locking in the P2 motif to yield the SAC attractor of the Phase Switch. Finally, the combined locking in of the P1, P5 and P6 motifs yields the G2 attractor.

### Stable motifs of the Phase Switch are conditionally stable motifs of the full cell cycle model

**Figure 4:**
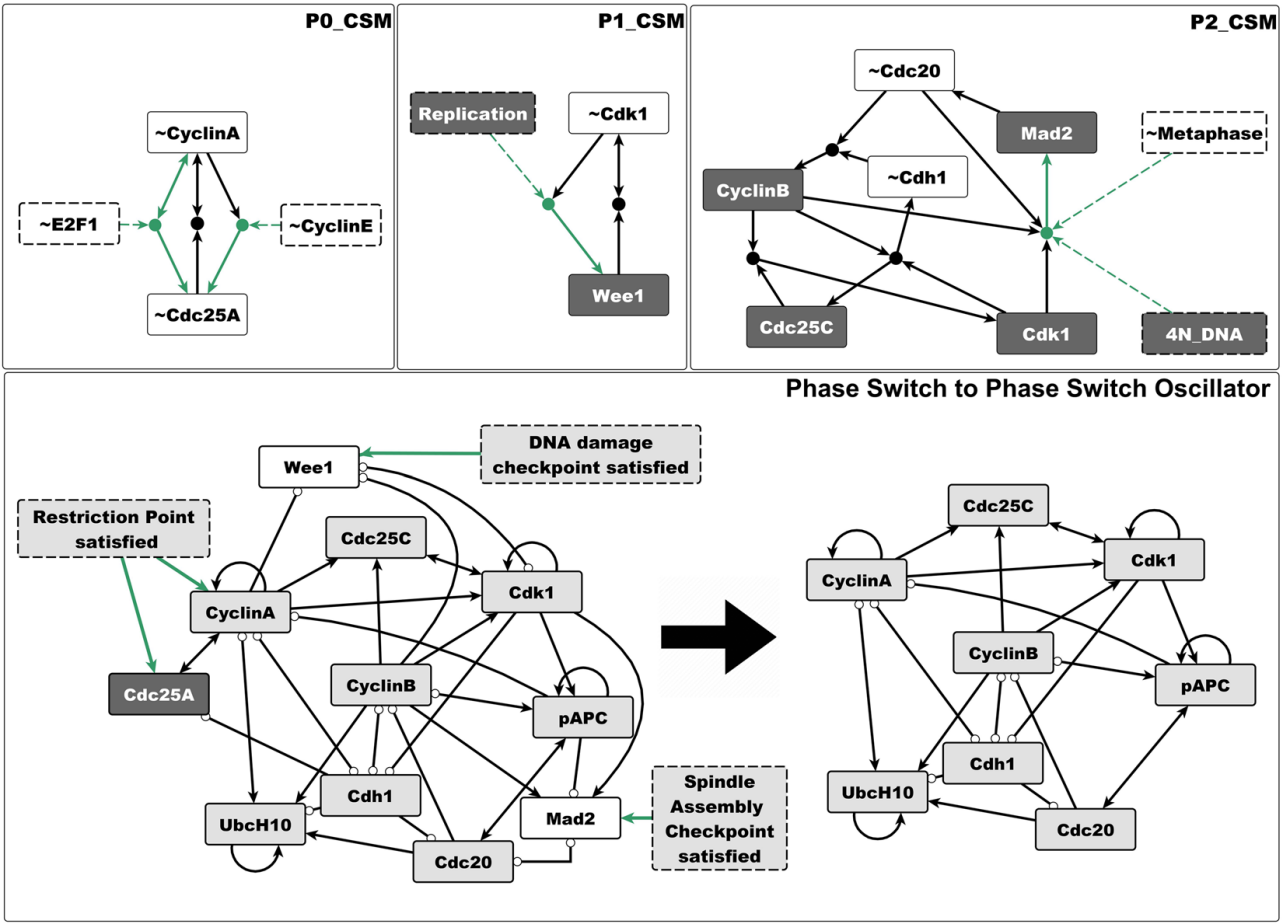
The stable motifs of the Phase Switch become conditionally stable motifs in the larger context of the cell cycle model. Due to the influences from the rest of the cell cycle network on Cdc25A, CyclinA, Wee1, and Mad2 (shown with green lines), the P0 motif turns into two conditionally stable motifs which differ only in their condition (~E2F1 or ~CyclinE, respectively, see top left panel). The P1 motif can only stabilize if the abstract node Replication is ON. P2 can only stabilize if the abstract regulator Metaphase is OFF and 4N DNA is ON simultaneously (top right panel). The Phase Switch Oscillator (bottom right) is obtained from the Phase Switch (bottom left) by assuming that the restriction point, DNA damage checkpoint and spindle assembly checkpoint are satisfied, which imply that Wee1 = Mad2 = 0 and Cdc25A = 1. As in Fig. 3, dark grey node background indicates the ON state of the node and white background refers to the OFF state.

In the full cell cycle model^17^ the nodes E2F1 and CyclinE of the Restriction Switch and the three abstract nodes (Replication, Metaphase, and 4N-DNA) regulate four nodes of the Phase Switch, namely Cdc25A, CyclinA, Wee1 and Mad2 (see Fig. 1 and Supplementary Notes S1–S3). Because of these incident influences, the stable motifs of the Phase Switch are only conditionally stable in the context of the larger model. The stabilization of the P0 motif requires the OFF state of either E2F1 or CyclinE (see P0_CSM on Fig. 4). The stabilization of the P1 motif requires the ON state of the Replication node, while the stabilization of P2 requires the simultaneous OFF state of Metaphase and ON state of 4N DNA. All of these nodes are in their required states for only specific phases of the cell cycle; for example E2F1 and CyclinE are OFF in the uncommitted state of the Restriction Switch. Because of this dependence on external regulators that are only transiently in the state necessary for stabilization, the P0, P1, and P2 motifs of the Phase Switch cannot stabilize permanently. In a dividing cell, the period during which one of these motifs maintains its stability corresponds to a cell cycle checkpoint: the P0 motif is stable before the restriction point (when E2F1 and CyclinE are inactive), the P1 motif is stable before the cell passes the G2 DNA damage checkpoint, and the P2 motif is stable before the cell passes the spindle assembly checkpoint^55^. Passage of each checkpoint changes the inputs to the Phase Switch such that the corresponding motif becomes unstable.

To explore what alternative behaviors remain in the attractor repertoire of the Phase Switch, we consider an extreme scenario with respect to stabilization. Namely, we assume that all three checkpoints are satisfied, which causes the stabilization of Cdc25A, Wee1 and Mad2 in the state *opposite* of their states in the stable motifs of the Phase Switch (see left side of bottom panel of Fig. 4), and thus destabilizes all three stable motifs. The resulting system, shown on the right side of the bottom right panel of Fig. 4, operates without any checkpoint control. This circuit shares some similarities with the network responsible for cell cycle progression in mammalian embryonic stem cells, in that it doesn’t have a restriction point^56^. In embryonic stem cells, E2F1, Cyclin E/Cdk2 and Cdc25A are continuously active in a cell cycle-independent manner^57^, allowing these cells to cycle continuously^58^. Contrary to the network driving embryonic stem cell division, the small circuit on Fig. 4 (right side of bottom panel) also lacks a DNA damage and spindle assembly checkpoint. Thus, the checkpoint-free version of the Phase Switch captures the functioning of the cell cycle when (and only when) *all* checkpoints are cleared without difficulty or pause.

After locking Cdc25A ON, and Wee1 and Mad2 OFF, the Phase Switch module is reduced to eight nodes and 28 edges. It is still strongly connected and contains positive and negative cycles of various lengths (right side of bottom panel of Fig. 4). Destabilizing the stable motifs of the Phase Switch might be expected to create a set of complex attractors, some of which may be dependent on the update scheme (see Supplementary Note S4). Interestingly, we find a single limit cycle attractor of 9 states using synchronous update (Supplementary Fig. S2), and a single 141-state complex attractor under asynchronous update. All remaining states (out of the 2^8^ = 256 states of this 8-node system) converge to the limit cycle/complex attractor. This shows that this dynamical system’s long-term behavior is a sustained oscillation. In recognition of this fact we refer to this modified system as the Phase Switch Oscillator (PSO).

### The Phase Switch Oscillator traces a robust cyclic trajectory through the three attractors of the Phase Switch

**Figure 5:**
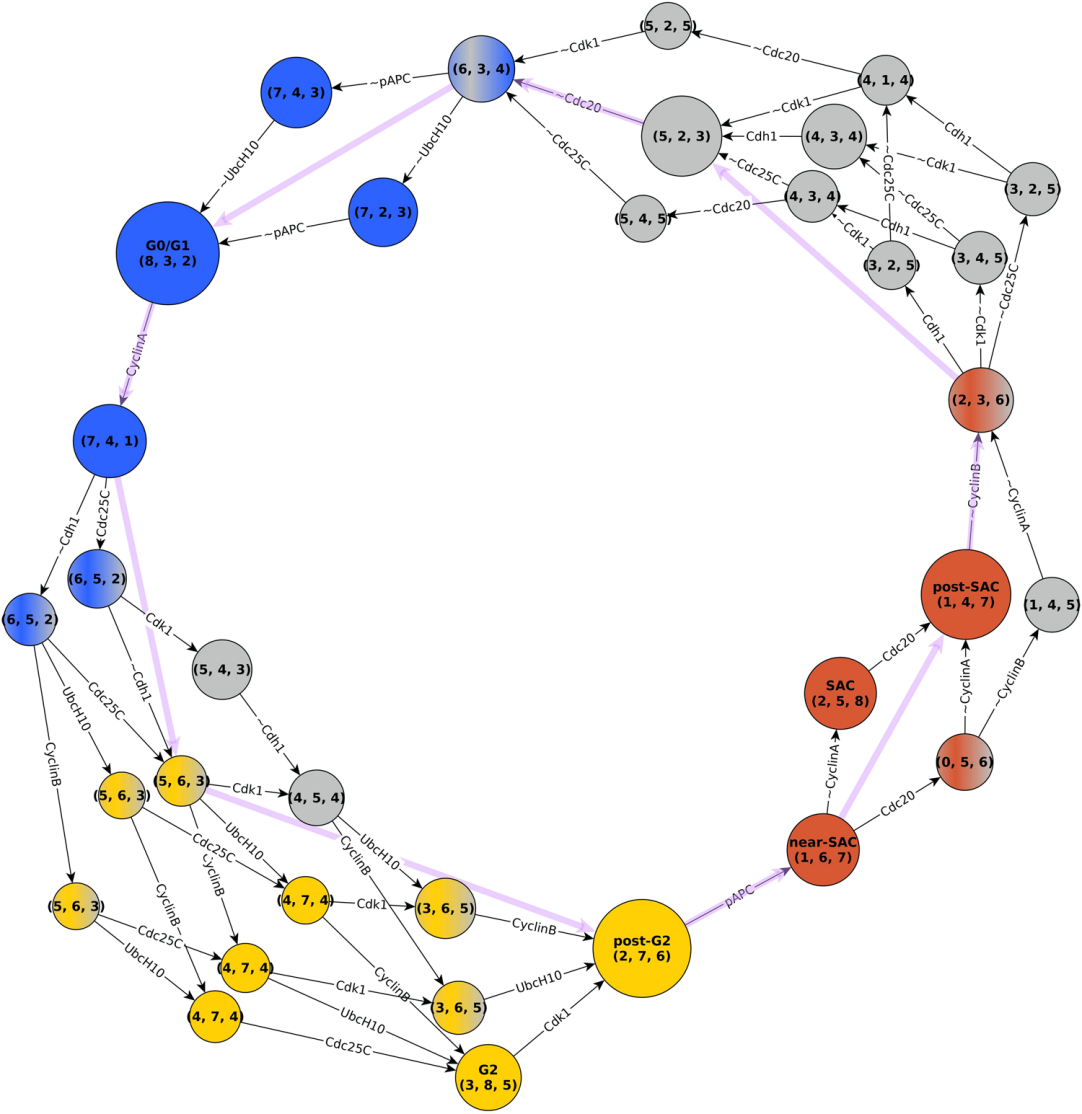
State transition graph (STG) representation of the complex attractor of the Phase Switch Oscillator. Each circle represents a state of the Phase Switch Oscillator, which is made up by the states of all the 8 nodes. The node sizes represent the visitation probabilities of the corresponding states (see Methods). States with visitation probability less than 0.5% are omitted. To provide a clear identifier without indicating the state of each node, each state of the system is labeled with its overlap with the three Phase Switch attractors, in the order G0/G1, G2, SAC (see Methods). If a system state overlaps one of the three attractors in 7 or 8 node states (more than 87% overlap), the node is colored with the color representing the relevant attractor, namely blue for G0/G1, yellow for G2 and brown for SAC. System states that have an overlap of less than 6 with an attractor are shown in grey. If the overlap is 6 the state is colored with a combination of grey with the color of the respective attractor. The color combinations mirror the transitions between the phases. Some important states are marked by a label representing the closest phenotype. Each edge label shows the node that changes state in the respective transition; if the node name is preceded by ~the node turns OFF, otherwise it turns ON. For simplicity we omit the self-loops that correspond to the cases where a node state is re-evaluated but does not change. The state transitions corresponding to synchronous update are shown in purple.

To evaluate to what extent the PSO’s long-term dynamics depend on stochasticity, we sampled the most frequently visited states of the complex attractor corresponding to general asynchronous update (see Methods), and overlaid the synchronous limit cycle on the resulting state transition graph (Fig. 5). We found that the PSO’s synchronous and asynchronous attractor follow similar paths along the cell cycle. Both pass through a state in which all the nodes of the Phase Switch are inactive, except for Cdh1; this is reflective of a quiescent cell or the early G1 phase of a cycling cell. Both trajectories also visit a state wherein CyclinA, CyclinB, and Cdk1 are ON. Cells in this state have just cleared the G2 DNA damage checkpoint (hence Cdk1 is active), but have not yet moved on to the SAC. Thus we denote it “post-G2”. In contrast, the state corresponding to a G2-arrested cell in which Cyclin A and B are expressed but Cdk1 is not yet ON (denoted G2) is only visited by one of three robust paths along the asynchronous complex attractor. The synchronous limit cycle updates three nodes in parallel during this step, and thus skips over the G2-arrested state. Following the post-G2 state, both attractors go through a state preceding the spindle assembly checkpoint wherein CyclinA has not yet degraded (which we denote “near-SAC”), as well as a state where Cdc20 has already turned OFF (which we denote “post-SAC”). Here too, the state closest to that of a cell arrested at the SAC (thus matching the SAC attractor of the Phase Switch) is only visited by the complex attractor; Cyclin A degradation and Cdc20 activation co-occur in the synchronous model. Supplementary Table S2 lists the state of all nodes in these labeled states.

The close agreement between the asynchronous complex attractor and the synchronous limit cycle is surprising because consecutive states of the synchronous state transition graph differ in up to three node states (multiple nodes can change state during one synchronous update), while the edges of the asynchronous state transition graph always represent changes in a single node (indicated as edge labels in Fig. 5). The loss of synchronicity between node state changes could have induced a dramatic departure from the synchronous limit cycle, as was observed in a previous Boolean model of the mammalian cell cycle by Fauré *et al*.^12^; only after partially restoring synchronicity did the Fauré *et al*. model yield near-cyclic trajectories. Remarkably, in the case of the PSO the vast majority of the paths in the asynchronous attractor follow the synchronous limit cycle and robustly adhere to its temporal ordering of states. Supplementary Fig. S3 compares the distribution of consecutive ON and OFF durations of each node with the case of a cycle in which each node switches state twice. Supplementary Note S5 provides an edge-by-edge comparison of the synchronous limit cycle and the asynchronous complex attractor, underscoring their agreement as both faithfully follow the order of phases in the cell cycle.

**Figure 6:**
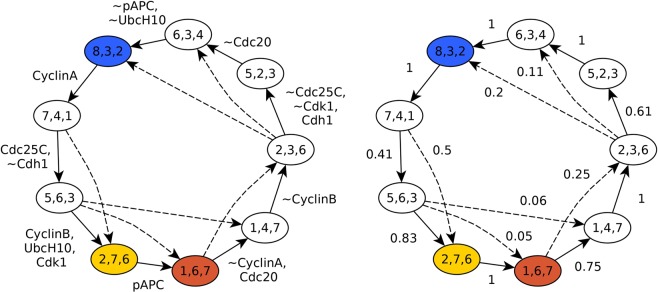
The shared backbone of the synchronous limit cycle and asynchronous complex attractor. The nodes represent the nine states of the synchronous limit cycle (the nodes connected by purple edges in Fig. 5); the node labels indicate the overlap of the corresponding state with the three Phase Switch attractors (in the order G0/G1, G2, SAC). The solid edges represent single state transitions obtained by synchronous update. These state transitions also appear in the asynchronous state transition graph, either as edges or as paths. The dashed edges indicate cases where paths exist in the asynchronous state transition graph that skip a state visited by the synchronous state transition graph. The states marked in blue, yellow and brown indicate the states closest to the G0/G1, G2, and SAC attractors, respectively. In the left panel the edge labels indicate the nodes that change state during the corresponding transition; nodes whose name is prefaced by ~turn OFF, the rest turn ON. For each synchronous state transition the asynchronous state transition graph contains a path that corresponds to sequential state changes of the same nodes. In the right panel the labels on the state transition edges indicate the probability of the state transition when using asynchronous update. State transitions with a probability less than 0.05 are omitted from this figure. Supplementary Table S3 indicates the probability of every possible transition between these nine states.

Figure 6 compactly summarizes this agreement in a “backbone” representation of the complex attractor. In order to quantify how closely the asynchronous dynamics adheres to the synchronous cycle, we computed the aggregated probability of all paths of the asynchronous state transition graph (STG) that start and end at the states of the synchronous limit cycle without visiting other states of the limit cycle (see Methods); these probabilities are indicated as edge labels in the network on the right panel in Fig. 6. Asynchronous update paths between limit cycle states that are not adjacent in the synchronous STG are deemed “shortcut transitions”; these transitions mix the node state changes involved in multiple steps of the synchronous update. A comprehensive list of the probability of all shortcut transitions is given in Supplementary Table S3. In spite of the random update order of the asynchronous update, only six shortcut transitions have probability higher than 0.05. In conclusion, despite the large variability of possible trajectories in a general asynchronous system, the emergent cycle of activations and deactivations in the PSO is remarkably deterministic.

### The Phase Switch Oscillator contains a cycle of conditionally stable motifs that sequentially stabilize each other and cause their own destabilization

To understand what causes the robustness of the oscillation and the sequential approach of the Phase Switch attractors we analyze the expanded network of the PSO, which encapsulates both the topological and logical features of the regulatory network. The details of this analysis are described in Supplementary Note S6. We find that the whole expanded network is an oscillating motif: it is strongly connected, it is composite-closed, it contains the complementary of each virtual node, and it does not have any stable motifs. While all virtual nodes participate in this oscillating motif, their contributions to the connectivity of the oscillating motif are not equal: CyclinA, pAPC and Cdh1 are the strongest contributors and Cdc25C, ~Cdc25C and ~UbcH10 are the weakest (see Supplementary Note S6). Parsing the logic sufficiency and necessity relationships embodied in the expanded network explains the trajectories of the state transition graph (see Supplementary Note S8 for a detailed comparison of the expanded network and the complex attractor).

**Figure 7:**
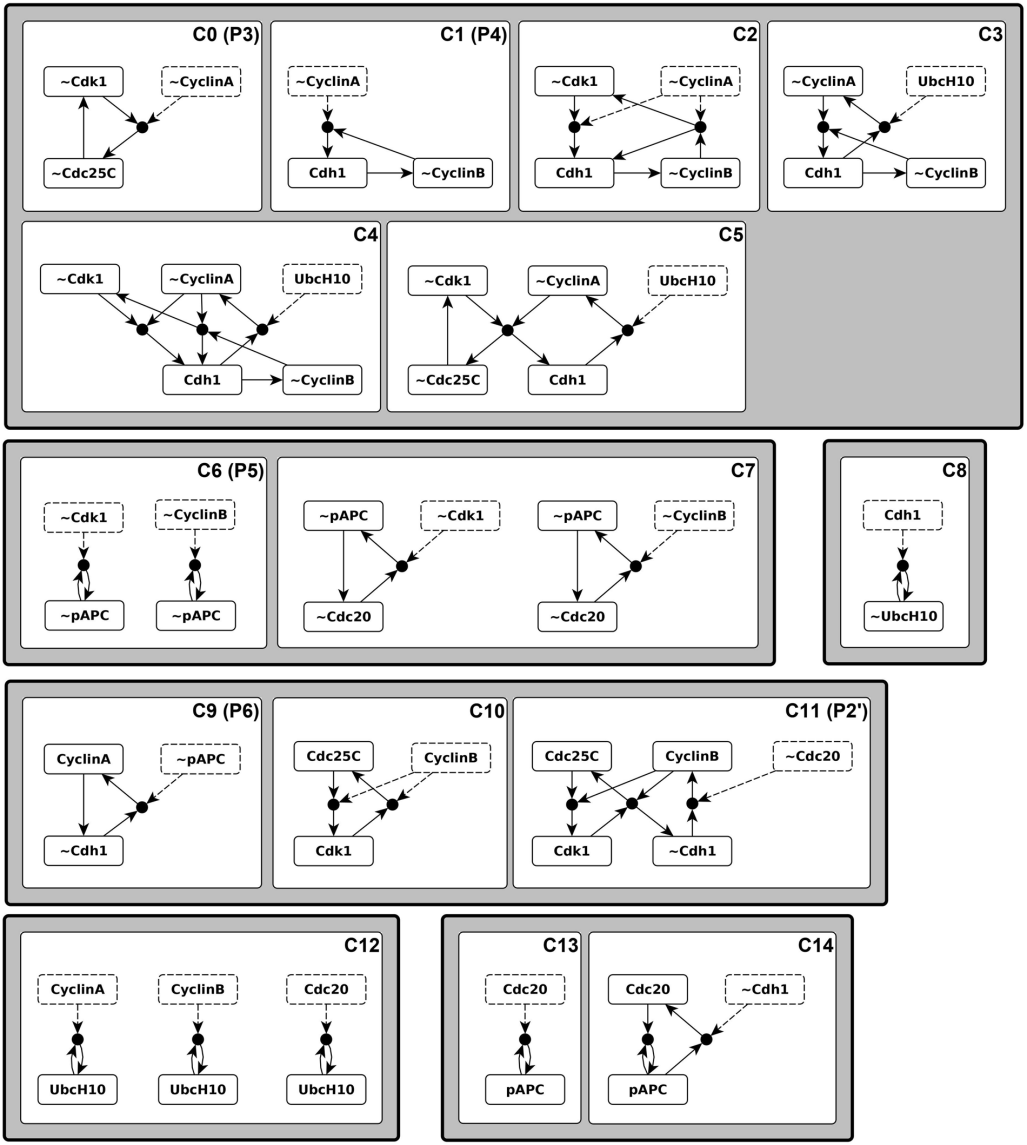
Conditionally stable motifs of the Phase Switch Oscillator (PSO). Conditionally stable motifs are represented here as subgraphs of the expanded network of the PSO. Virtual nodes with dashed outline represent the conditions. The labels in the top right corner of the white boxes indicate the name of the conditionally stable motif as well as the corresponding Phase Switch motif. Multiple motifs in the same white box (e.g. C7) consist of the same virtual nodes but with different conditions. The grey boxes around groups of white boxes illustrate that CSMs with shared states naturally fall into groups.

The fact that the whole expanded network is a single oscillating motif explains why the PSO does not have point attractors, but by itself does not explain why there is a single complex attractor and why it is so close to a cycle. As a next step toward answering these questions, we identified all conditionally stable motifs (CSMs) in the PSO that have a single condition (see Methods). These are depicted and labeled in Fig. 7. The smallest CSM is a node that can maintain its state with the help of another node. This situation appears in the expanded network as a virtual node that has an edge pointing to a composite node and receives an edge from the same composite node. The composite node’s other regulator serves as the condition for the CSM. There are two such nodes, pAPC and UbcH10; both virtual nodes of each form CSMs (C6, C8, C12 and C13 in Fig. 7). Other elementary CSMs of the Phase Switch Oscillator contain two or three virtual nodes that form one or more cycles. Several elementary CSMs overlap in yet larger CSMs, indicating that satisfying a single condition can often stabilize relatively large subnetworks.

Four CSMs of the Phase Switch Oscillator coincide with the four CSMs of the Phase Switch: C0 is the same as P3, C1 coincides with P4, C6 is P5, and C9 coincides with P6, respectively (compare Fig. 3 with Fig. 7). The CSM C11 includes four of the five virtual nodes of the P2 stable motif (only Mad2, which is not present in the PSO, is absent); thus, the C11 (P2’) notation. This preservation of conditionally stable motifs indicates that the PSO’s dynamic trajectory can at least transiently visit the attractors of the Phase Switch. In addition, the PSO has CSMs that are not present in the Phase Switch; notably C14, which contains pAPC = 1 together with the Cdc20=1 state absent from all three attractors of the Phase Switch. The activation of this conditionally stable motif represents the activation of the pAPC/Cdc20 complex, which starts chromosome separation^59^.

**Figure 8:**
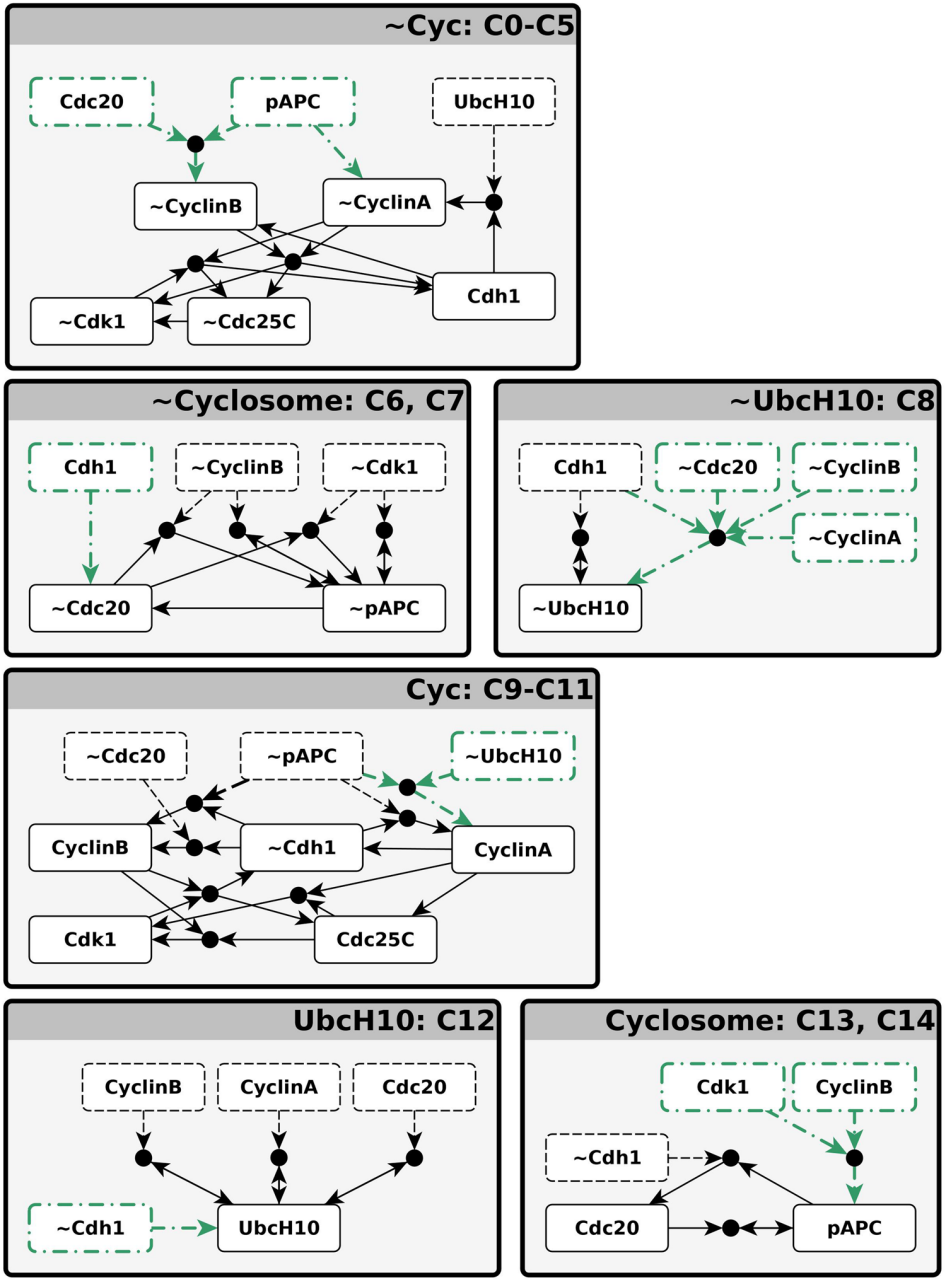
The logical relationships between conditionally stable motifs (CSMs) of the Phase Switch Oscillator. The fifteen single-condition CSMs fall into six groups of overlapping CSMs, indicated by the boxes labeled Cyc, ~Cyc, Cyclosome, ~Cyclosome, UbcH10, and ~UbcH10. The union of the CSMs in each group is depicted as a subgraph of the expanded network within each of the boxes. The box labels list the CSMs that are merged in each case; the six CSM groups correspond to those of Fig. 7. Within each box, black dashed lines indicate the conditions of the merged CSM and the green dash-dotted lines indicate regulation external to CSMs (not required for its stabilization). Importantly, sustained activity of each row of boxes leads to activation of the next row (with the last row looping back to the first).

Inspection of the single-condition CSMs in Fig. 7 suggests merging multiple overlapping CSMs into larger CSMs. One large group unites conditionally stable motifs C0-C5 and is composed of the virtual nodes ~CyclinA, ~CyclinB, ~Cdk1, ~Cdc25C, and Cdh1. Strikingly, these virtual nodes’ complementary nodes, i.e., CyclinA, CyclinB, Cdk1, Cdc25C, and ~Cdh1, also form an overlapping group of conditionally stable motifs, namely C9-C11 (See also Supplementary Fig. S4). We denote the CSM composed of this latter group of virtual nodes “Cyc”, as it corresponds to the activation of the key cyclins. Similarly, we name the CSM formed by the merger of C0-C5 “~Cyc”, as it represents the inactivation of the key cyclins and is complementary to the Cyc CSM. The overlapping CSMs C6 and C7 can also be merged to create a larger CSM that contains the virtual nodes ~Cdc20 and ~pAPC. This merger has a complementary counterpart in the union of the C13 and C14 CSMs. We call the C13-C14 merged CSM “Cyclosome” in reference to the Cdc20-bound APC/C complex known as the cyclosome. Its complement, the C6-C7 merger, is called “~Cyclosome”. Finally, the various forms of C12 can be merged to form a larger CSM containing only UbcH10 as a state, while the CSM C8 acts as the complementary CSM. As these contain only UbcH10 and ~UbcH10 as states, we refer to these CSMs by the names “UbcH10” (C12) and “~UbcH10” (C8). We note that these six merged CSMs are the six largest CSMs in the expanded network (see Methods). The merged CSMs and their regulators are depicted in Fig. 8 as expanded networks. Each of the six boxes in Fig. 8 graphically indicates the regulatory functions for each node of the corresponding CSM. Thus, each box indicates the states of the regulators for which all the nodes of the CSM will achieve their corresponding state. Regulators whose states serve as conditions of the CSM are shown in dashed outline, while regulators that are outside the CSM (able to influence it but not *necessary* for stabilization) are shown in dash-dotted outline. We wish to emphasize that the groups of virtual nodes we named Cyc, Cyclosome, UbcH10 and their negated counterparts ~Cyc, ~Cyclosome and ~UbcH10 are not arbitrary, but emerged naturally from the structure of the expanded network as the largest disjoint CSMs.

An important feature shared by several CSMs of the Phase Switch Oscillator is that they cause the deactivation of their own conditions. In other words, sustained activity of the virtual nodes in these CSMs eventually leads to the activation of virtual nodes that contradict the states of the CSM conditions (see Supplementary Note S7 for examples). The merged CSMs have the same self-destabilizing properties. For example, by examining Fig. 8, one can determine that sustained activity of the ~Cyc CSM (C0-C5) eventually leads to activation of the ~Cyclosome CSM (C6-C7) because the five virtual nodes within ~Cyc contain the two conditions (nodes with dashed outlines) and the external regulator (in dash-dotted outline) necessary to activate the two virtual nodes of ~Cylosome. The five virtual nodes within ~Cyc are also sufficient to activate ~UbcH10 (C8). The CSMs ~Cylosome and ~UbcH10 contradict all the conditions and external regulators of ~Cyc. Indeed, the sustained activity of ~Cyclosome and ~UbcH10 eventually lead to the activation of Cyc, which contradicts ~Cyc in every virtual node. Similar relationships exist between each row of Fig. 8 and the row below it (or between the bottom row and the top row).

### A higher-level network of three nodes qualitatively replicates the oscillation

It is possible to make the relationships between the six CSMs of Fig. 8 (see also Supplementary Fig. S4) precise by designating a new Boolean variable for each complementary pair of the merged CSMs. Each of the six CSMs can thus be viewed as a virtual node, corresponding to one of two states of a corresponding meta-node. We label these three meta-nodes Cyc, Cyclosome, and UbcH10. The higher order logic of the CSM meta-nodes can be distilled into the regulatory functions

$$\begin{array}{rcl}{{\rm{f}}}_{{\rm{Cyc}}} & = & ({\rm{not}}\,{\rm{Cyclosome}}\,{\rm{and}}\,{\rm{Cyc}})\,{\rm{or}}\,({\rm{not}}\,{\rm{Cyclosome}}\,{\rm{and}}\,{\rm{not}}\,{\rm{UbcH10}})\\ {{\rm{f}}}_{{\rm{Cyclosome}}} & = & {\rm{Cyc}}\\ {{\rm{f}}}_{{\rm{UbcH10}}} & = & {\rm{Cyc}}\,{\rm{or}}\,{\rm{Cyclosome}}\,{\rm{and}}\,{\rm{UbcH10}}\end{array}$$

**Figure 9:**
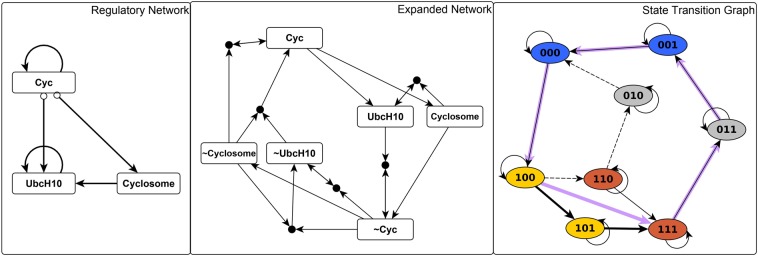
The logical relationships that determine the transitions of the PSO can be effectively illustrated by defining aggregated meta-nodes for overlapping conditionally stable motifs. The Cyc meta-node contains CyclinA, CyclinB, Cdc25C, Cdk1, and ~Cdh1. The Cyclosome meta-node includes the virtual nodes pAPC and Cdc20. The complementary node (negation) of a meta-node includes the complementary nodes of the meta-node’s constituent virtual nodes. The first two panels indicate the regulatory and expanded network of meta-nodes. The panel on the right shows the state transition graph of the meta-node network. In this panel the labels of each state are in the order Cyc, Cyclosome, UbcH10. Node background color indicates the states closest to the attractors of the Phase Switch: blue for G0/G1, yellow for G2, brown for SAC. The synchronous cycle is shown by purple edges. The asynchronous complex attractor is made up by two cycles of unequal size, but each of which approaches the three attractors in the same order. The difference between the two cycles is whether UbcH10 activates and then deactivates (longer cycle) or stays inactive (short cycle with dashed edges). This latter process has a very low probability.

These regulatory functions express the following logical relationships: Cyclosome and UbcH10 inactivate Cyc; the only possibility for Cyc activation is the simultaneous inactivity of both Cyclosome and UbcH10. Existing Cyc activity can be maintained if Cyclosome is inactive. Cyc is sufficient for the activation of Cyclosome and UbcH10. UbcH10 activity can also be sustained if Cyclosome is active. Figure 9 illustrates the regulatory and expanded networks of meta-nodes as well as the corresponding STG. The resulting regulatory network relating the three meta-nodes preserves certain structural properties of the regulatory network of the PSO. Most importantly, all negative feedback loops between nodes in the PSO are represented as negative feedback loops between meta-nodes, while each positive feedback loop in the PSO is internal to exactly one meta-node (or is represented explicitly as a positive self-loop).

The higher-order regulatory logic of the CSM meta-nodes explains the PSO’s cycling between states close to the G0/G1, G2, SAC attractors, as shown in the right panel of Fig. 9 (see also Supplementary Note S8). The G0/G1 state corresponds to the inactivity of all three meta-nodes. This is not an attractor; inactivity of pAPC and Cdc20 implies that Cyc will activate. When this is achieved, the system is closest to the G2 attractor of the original Phase Switch. Next, the activity of Cyc drives the activation of UbcH10 and Cyclosome in a stochastic order. The probability of UbcH10 turning on before Cdc20 and pAPC, and thus activating the UbcH10 meta-node before the Cyclosome meta-node, is at least 0.98 (see Supplementary Table S3). The rare case in which Cyclosome activates before UbcH10 leads to a path of state transitions (shown with dashed lines) from a G2-like state to the G0/G1 state without ever activating UbcH10, consistent with the observation that fixing UbcH10 off preserves the complex attractor. In the more likely scenario, UbcH10 activates first, and then the activation of the Cyclosome node (pAPC and Cdc20) marks the spindle assembly checkpoint. This state is also short-lived, as Cyc is deactivated by Cyclosome, which in turn causes the inactivation of both Cyclosome and UbcH10. Thus, the system returns to the G0/G1 state.

### Examining the motif structure and dynamics of networks with a locked node reveals the differences between the nodes’ influence

**Figure 10:**
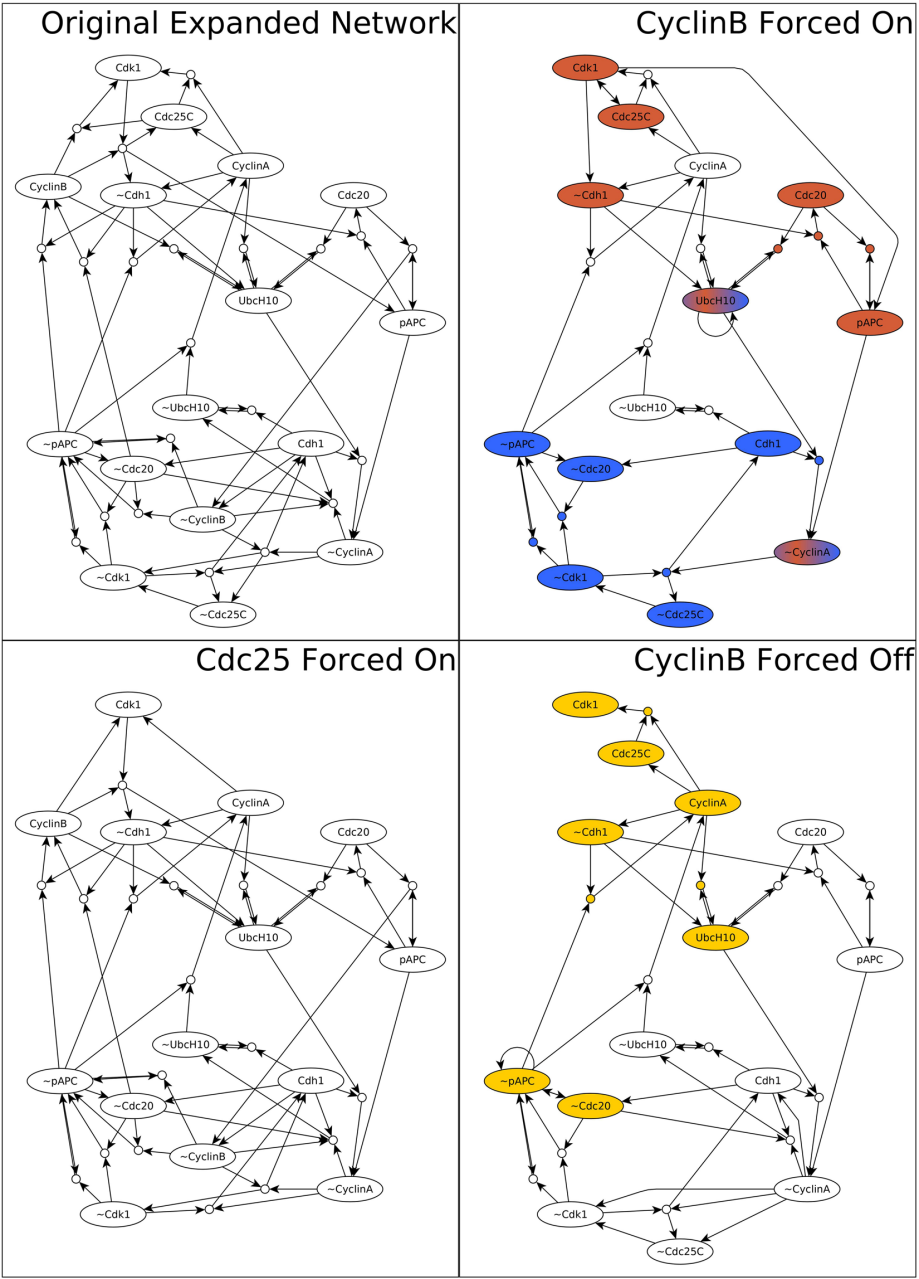
The expanded network that results from no intervention (top left panel) or three characteristic interventions (other panels as indicated by panel titles). Virtual nodes whose label is the node name preceded by ~indicate the off state of the relevant node. The three interventions exemplify each of the three attractor categories outlined in the main text: retained oscillation (bottom left, UbcH10 off), single point attractor (bottom right, CyclinB off), and multiple point attractors (top right, CyclinB on). The group of virtual nodes that make up each point attractor are highlighted in color. The blue state has the greatest overlap with the G0/G1 attractor of the Phase Switch, the yellow state most closely overlaps with the G2 attractor, and the brown state is most similar to the SAC attractor.

All eight nodes of the Phase Switch Oscillator participate in the oscillation and spend a similar amount of time in their two states (see Supplementary Fig. S3). All 16 virtual nodes participate in at least one CSM, but their contribution to the connectivity of the expanded network is not equal (see Supplementary Note S6). We next asked whether all nodes contribute equally to the oscillation. To evaluate each node state’s contribution to the complex attractor, we systematically set each node in its active or inactive state and identify the motif structure and attractor repertoire of the thus-modified dynamical system. As described in detail in Supplementary Note S9 and Supplementary Table S4, the modified systems’ dynamic behaviors fall into three categories: (1) in three cases the PSO oscillation is preserved as the sole attractor, (2) in 11 cases the modified system has a single point attractor, and (3) in two cases the modified system has multiple point attractors. The expanded networks for representatives of each of the three categories are shown in Fig. 10, alongside the original system’s expanded network.

The most interesting example is Cyclin B, whose locking ON creates two highly dissimilar fixed point attractors (see right panels of Fig. 10). The bistability in the presence of forced CyclinB expression is due to the fact that sustained CyclinB is the (direct or indirect) condition for two mutually exclusive CSMs (C10 and C12). In contrast, when CyclinB is held inactive the CSMs C7 and C9 can stabilize, resulting in a single point attractor most similar to the G2 attractor (see Supplementary Note S9). Thus, control of CyclinB can yield any of the three attractors of the Phase Switch (i.e., the states at checkpoints of the cell cycle), which is consistent with biological knowledge^45,46,55,60^. Moreover, enforced step-wise changes between fixed states of CyclinB can induce attractor transitions that mimic cell cycle progression (as illustrated in Supplementary Fig. S5 using general asynchronous update). The constraint CyclinB = 0 drives the Phase Switch Oscillator to a unique G2-like state in all update schemes. This state is in the basin of attraction of the SAC-like attractor of the CyclinB = 1 constrained PSO (in particular, the C10 conditionally stable motif is active). Thus, if CyclinB is absent until a steady state is reached, and is then reintroduced, the model system undergoes a transition from a G2-like state to a SAC-like state. This model behavior matches experimental observations, as introduction of Cyclin B to frog oocytes with replicated DNA but no cyclin expression was shown to drive these cells into mitosis^45,46,60^. Furthermore, if we remove CyclinB *after* the SAC-like state is reached, then the system passes through a G0/G1-like state^47,61,62^, which was not visited by the trajectory from the G2-like state to the SAC-like state (Supplementary Fig. S5). A similar hysteresis in response to the increase vs. decrease of CyclinB was experimentally observed in Xenopus embryonic cells^45^. In conclusion, we predict that there exists a sequence of repeated changes in CyclinB that can drive the system to visit the attractors of the Phase Switch in the same order as the cell cycle.

## Discussion

Here we presented follow-up analysis of a Boolean model of the mammalian cell cycle which integrates previously published models^6–8,12,41,42^ to synthesize the regulatory logic of the cell cycle control machinery. This model agrees with continuous (ODE-based) models in recognizing the importance of bistable switches (based either on mutual activation or mutual inhibition) in this regulatory logic. As beautifully illustrated in a recent review article by Novák *et al*.^63^, these switches are concatenated and nested. Matched pairs of mutually inhibitory bistable switches underlie the cell cycle checkpoints. Once the pair of switches is toggled, the transition through the checkpoint is irreversible. The logic-based methods we present here offer a related and complementary way to understand the attractor repertoire that arises from coupled and nested bistable switches. The concept of stable motif expresses a stable switch state. Thus, the activation of a stable motif marks a point of no-return in a system’s trajectory. Here we introduced conditionally stable motifs, which can maintain a fixed state of their constituent nodes as long as the state of one or more nodes external to the motif is maintained. Intuitively, conditionally stable motifs are reversible switches that can maintain their state when their conditions are fulfilled, but are reversed when their conditions are violated. Our analysis focused on understanding the connection between reversible switches and the cyclic activation and deactivation of cyclins during the cell cycle. To do this, we considered a cell that lacks the restriction, DNA damage and spindle assembly checkpoints. Our results indicate that the negative feedback loop formed by a group of strongly coupled switches (encompassing CyclinA, CyclinB, Cdk1, Cdh1, Cdc25C), on the one hand, and the complex of pAPC and Cdc20, on the other hand, is a main contributor to this cyclic behavior.

Conditionally stable motifs are useful generalizations of stable motifs. Stable motifs are well-defined within the context of a model. Yet, as all models are ultimately incomplete, it is possible that a more complete model would have additional regulators that transform the stable motif into a conditionally stable motif. Through our analysis of the Phase Switch and the Phase Switch Oscillator we have uncovered three key features of conditionally stable motifs (CSMs). First, they can play an important role in the decision-making of multi-stable systems. For example, in the Phase Switch module the stable motif P1 is compatible with both G0/G1 and G2 attractors. The subsequent stabilization of the conditionally stable motif P5 and P0 or, alternatively, P5 and P6, steers the system into one or the other attractor respectively (see Fig. 3). Second, in systems with complex attractors, CSMs reduce noise introduced by stochastic update order by temporarily eliminating degrees of freedom. Indeed, CSMs with the fewest conditions capture the temporary stability of short-range positive feedback loops, which temporarily fix the states of the nodes in the feedback loop. Third, oscillation requires that no CSM has stabilized conditions, and therefore the pattern of CSM condition activation and deactivation can illuminate the nature of the oscillation.

Both the Phase Switch and Phase Switch Oscillator contain induced strongly connected subgraphs that are sign-consistent (i.e., lack negative feedback loops and incoherent feedforward loops). For example, the nodes pAPC and Cdc20 and the two positive edges between them form one such sign-consistent, strongly connected subgraph (see Fig. 4). A significant body of work applied to both continuous and discrete dynamical systems indicates that sign-consistent systems (also called monotone systems) have highly predictable and ordered dynamics^64^. Even though the Phase Switch and Phase Switch Oscillator are not sign-consistent, studying the connections between their largest sign-consistent subgraphs is a fruitful way forward. In the specific case of the Phase Switch Oscillator, the largest sign-consistent subgraph is the group made up by CyclinA, CyclinB, Cdk1, Cdh1, and Cdc25C, a group whose two opposing states make up the two largest conditionally stable motifs, Cyc and ~Cyc. The second-largest sign-consistent subgraph is formed of pAPC and Cdc20, and determines the Cyclosome and ~Cyclosome CSMs. There are three negative feedback loops in the Phase Switch Oscillator: one between CyclinA and UbcH10, one between CyclinB and pAPC, and one between CyclinA, Cdk1 and pAPC (see Fig. 4). Merging the sign-consistent, strongly connected subgraphs into single meta-nodes yields the coarse-grained network of Fig. 9. This network merges the latter two negative feedback loops into a negative feedback loop between the Cyc and Cyclosome meta-nodes. Studying the general relationship between the largest sign-consistent subgraphs and largest CSMs is an interesting topic for future work.

Our analysis yields novel biological insights and predictions. For example, our analysis of the Phase Switch Oscillator with a locked-in state of CyclinB confirms the important role of CyclinB in driving the cell cycle of embryonic cells, and mitosis in somatic cells. We predict that there exists a sequence of repeated changes in CyclinB that can be used to drive the system to visit the attractors of the Phase Switch in the same order as the cell cycle (see Supplementary Fig. S5). More broadly, our findings support the conclusion that a combination of bistability and negative feedback underlies many biochemical oscillators^27,45^. Our newly introduced concept of conditionally stable motif may also be helpful in addressing biological learning and adaptation in a network framework^30^.

The expanded network framework is part of a broader effort to characterize and represent as a network the causal relationships between variables of a dynamical system. Related concepts include signed interaction hypergraphs^21^ and dynamics canalization maps^65^. For example, the logic domain of influence of a node state^66^ is a subgraph of the expanded network that is conceptually similar to the three-valued (0, 1, unknown) logical steady state that results from fixing a node state^21^ and to the dynamical modules of dynamics canalization maps, which represent the states inexorably stabilized by an input configuration^65^. The concepts of expanded network and stable motif have been generalized and implemented in multi-level discrete systems and continuous-variable systems described by ordinary differential equations^33,52^. When considered generally, the expanded network encodes causal links between regions of state-space. Each of its virtual nodes represents a region of state-space (e.g., the region in which a particular variable takes a specified value or range of values) and the composite nodes represent the intersection of the virtual node regions. Once an expanded network is constructed for a given dynamical system, be it discrete or continuous, the concept of a conditionally stable motif is immediately applicable. Thus, it is possible that by using the methods of Rozum & Albert 2018^52^, many of the concepts we have introduced here can be generalized to multi-level discrete dynamical systems and ODE models.

Our analysis indicates that the influences on the Phase Switch originating from the other modules become functional in a manner that allows the Phase Switch to approach one of its attractors (as the cell reaches the relevant checkpoint), but then they destabilize this attractor as the checkpoint is cleared. In other words, the network around the Phase Switch helps provide the *conditions* that govern its stable motifs. Nevertheless, we found that the robust channeling of its dynamics along a limit cycle is intrinsic to this network. Thus, the Phase Switch balances the need to stably maintain the cell at each checkpoint with the need for a robust limit cycle when checkpoints are cleared without issue. The methodology described in this paper can be used to understand the complex oscillation that emerges from the coupling of the Phase Switch and the Restriction Switch in the presence of growth factors. As explained in Deritei *et al*.^17^ and illustrated in the top right panel of Fig. 1, this attractor recapitulates the cell cycle in the presence of the checkpoints we removed in this study, while toggling the combinations of the module attractors. Analyzing the conditionally stable motifs of the coupled model could shed light on an even more comprehensive coarse-grained logical network that drives the cell cycle, and offer further insight on dynamical modularity.

## Methods

### Mapping the complex attractor

The trajectories generated by general asynchronous update can be interpreted as a random walk on the state transition graph. During a random walk on a directed network a walker on node $i$ at time step $t$ randomly chooses one of the edges going out of $i$ and traverses that edge in one time step. Using general asynchronous update every node has the same probability of being updated, thus every outgoing edge (i.e. every state transition that yields a different state than the starting state) has the same probability. An extended simulation of the model trajectory starting from any node in the basin of attraction of the complex attractor (i.e. the states that are starting points of trajectories that reach the complex attractor, which in the case of the PSO is the entire state space) gives us a reliable sample of the most probable states and transitions. We performed a random walk of 100,000 steps and determined the visitation counts of states and transitions. We then filtered the state transition graph, leaving only states that were visited at least 500 times. We validated the visitation probabilities emerging from our sampling process by using the PageRank algorithm^67^ on the full complex attractor. Mapping of the full state transition graph of the eight-node PSO is tractable; this would not be the case for larger networks.

### Representing a state of the phase switch oscillator by its overlap with the three attractors of the phase switch

The nodes of the state transition graph of the PSO represent vectors of the states of the 8 nodes of the PSO. For example, one such system state is “0,0,0,1,0,0,0”, in the order “Cdc25C, CyclinA, Cdk1, CyclinB, Cdh1, pAPC, Cdc20, UbcH10”. We use a more concise alternative notation that indicates the closeness of each state to the three attractors of the Phase Switch (indicated on Supplementary Table S1). We define the overlap of a system state with an attractor as the number of nodes whose state is the same in the system state and in the attractor. The Phase Switch has 11 nodes, 3 of which are locked in when defining the Phase Switch Oscillator. We do not consider these 3 nodes. As an example, the state “0,0,0,1,0,0,0” overlaps the G0/G1 attractor in all 8 states, it overlaps the G2 attractor in three states (those of Cdk1, pAPC, Cdc20) and it overlaps the SAC attractor in two states (those of CyclinA and Cdc20). The overlap triple of a system state indicates the overlap with the three attractors in the order (G0/G1, G2, SAC).

The previously mentioned state’s overlap triple is (8,3,2), meaning that it corresponds to the G0/G1 state. Similarly, the state that perfectly overlaps the G2 attractor of the Phase Switch has an 8 in the second position; we denote this state G2. The state that perfectly overlaps the SAC attractor has an 8 in the third position; we denote this state SAC. The overlap triple notation is not unique, for example, there are three states with overlap triple (4,7,4), but these states can be disambiguated based on the identity of the node state changes that yield the state or leave the state. For easier interpretation we introduce a few additional phenotypic notations on Fig. 5. We denote two intermediary states between the G2 state and the SAC “post G2” (this is a state that differs from the G2 state only in that Cdk1 has already turned ON) and “near-SAC” (in this state CyclinA has not turned OFF yet). The denote “post-SAC” the state that differs from the SAC state in that Cdc20 has turned ON.

### Coarse-graining the complex attractor based on established proxy states

On the state transition graph corresponding to general asynchronous update each outgoing edge has the same probability (as all node updates are equiprobable). We define the edge probability as the inverse of the out-degree of the edge’s source node.

Using the complete state transition graph corresponding to general asynchronous update, we determine the transition probability between the nine states of the synchronous limit cycle, which we termed proxy states. For each pair of proxy states, we find all simple paths between the two states that do not involve any other proxy state and calculate the probability of each path as the product of edge probabilities over the path. The transition probability between the two proxy states is the sum of the path probabilities. We use these probabilities to construct the backbone of the complex attractor: starting with a disconnected graph of proxy states, we consider each ordered pair of proxy states, determine the transition probability from the first to the second, and add a corresponding directed edge if the transition probability is larger than 0.05.

### Identification of conditionally stable motifs

Every CSM is a strongly connected component of the expanded network, and therefore it is either a cycle or can be viewed as a union of directed cycles. The latter follows from the fact that every node in a strongly connected component must have a path that leads to itself, and for every edge $(i,\,j)$ in the component, there is a path from node $j$ to node $i$. To satisfy the consistency criterion of the CSM definition, the corresponding cycle-set must be consistent in the sense that no virtual node or composite node in any of the cycles may be logically incompatible with any other node of any cycle in the set. Furthermore, the cycles must collectively form a strongly connected component, which precludes a set of disjoint cycles. The cycle sets subject to these two criteria (consistency and lack of a disjoint partition) are in one-to-one correspondence with the CSMs in the expanded network.

We leverage this fact to enumerate all CSMs. We begin by considering every self-consistent cycle in the expanded network. This is equivalent to considering every positive feedback loop in the regulatory network^31^. We define a network of cycles (which we call cycle graph) in the following way: (i) each node of the network is a cycle and (2) undirected edges are drawn between a pair cycles if they share a node in the expanded network and are mutually consistent (see Supplementary Fig. S6). If two cycles are connected, then there is a path from any node in one cycle to any node of the other, and so the union of the corresponding cycles is strongly connected. In the same vein, any connected subgraph of the cycle graph corresponds to a strongly connected component of the expanded network. If a subgraph in the cycle graph is not connected, then its components constitute a disjoint partition of the union of cycles. Therefore, every CSM node set corresponds to either a single cycle or a connected subgraph of the cycle graph. The reverse is not necessarily true: connected subgraphs of the cycle graph might not be composed of consistent cycles. Consistent connected subgraphs of the cycle graph, however, are in one-to-one correspondence with the CSMs of the expanded network.

In the PSO, the number of positive feedback loops is small enough that the connected subgraphs of the cycle graph can be exhaustively constructed (Supplementary Fig. S6). The computational complexity of our current implementation scales combinatorially in the number of positive feedback loops, which itself can increase dramatically with the number of nodes in the network. Development of more efficient or heuristic methods will be the focus of future work.

### Implementations

For synchronous and asynchronous simulation of the dynamic models of the Phase Switch and Phase Switch Oscillator we used the BooleanNet python library available at https://github.com/ialbert/booleannet. The identification of the stable motifs was done using the Java library available at https://github.com/jgtz/StableMotifs. The building of the expanded network based on the regulatory functions was done using the BooleanDOI python library available at https://github.com/yanggangthu/BooleanDOI. A descriptive supplementary Jupyter Notebook that reproduces our main computational results is available at: https://github.com/deriteidavid/conditionally_stable_circuits.

The notebook includes the following implementations (see Methods for details):

1. Creating a BooleanNet instance of the PSO model.
2. Identifying the synchronous attractor by simulation.
3. Sampling the complex attractor with general asynchronous update scheme, where the resulting state transition graph can be arbitrarily filtered and exported into a graphml object. This includes the comparison of the states with the Phase Switch attractors.
4. Determining the full state transition graph of the PSO and using the PageRank algorithm to validate the filtered sample.
5. Determining the ‘backbone’ of the complex attractor.
6. Generating the expanded network of the PSO.
7. Finding the conditionally stable motifs of the PSO.

## Supplementary information

Supplementary Tables

Supplementary Figures

Supplementary Notes 1-9

Supplementary Note S10
