# Supplementary material for: A feedback loop of conditionally stable circuits drives the cell cycle from checkpoint to checkpoint
Source: Sci Rep. 2019 Nov 11;9:16430. doi: 10.1038/s41598-019-52725-1 (PMC6848090; doi:10.1038/s41598-019-52725-1)

Supplementary Figures for

A feedback loop of conditionally stable circuits drives the cell cycle from checkpoint to checkpoint

Dávid Deritei[1,2], Jordan Rozum[1], Erzsébet Ravasz Regan[3], Réka Albert[1]

[1]Department of Physics, Pennsylvania State University, University Park, PA, United States of America
[2] Department of Network and Data Science, Central European University, Budapest, Hungary
[3] Biochemistry and Molecular Biology, The College of Wooster, Wooster, OH, United States of America

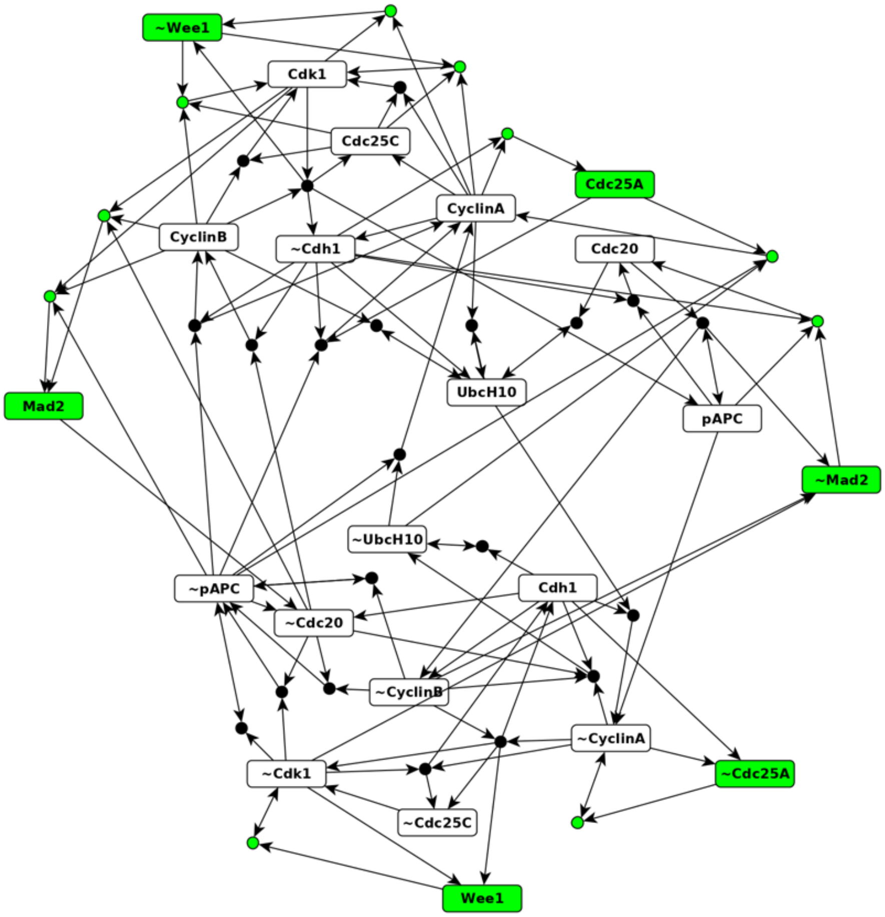

Supplementary Figure S1. The expanded network of the Phase Switch. The virtual nodes whose state is fixed in the Phase Switch Oscillator are shown in green.

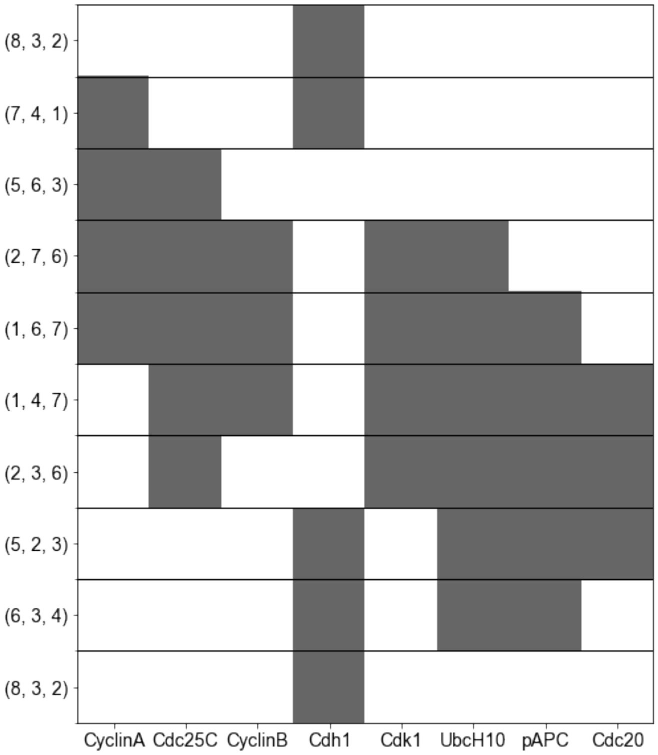

Supplementary Figure S2. The limit cycle of the Phase Switch Oscillator under synchronous update. Each column of squares indicates the states of the node written below the column. Each row corresponds to a state of the system. To use an identifier that is more economical than indicating the state of all 8 nodes, we describe the state by its overlap with the three attractors, in the order (G0/G1, G2, SAC); see Methods for a more detailed description. Each pair of successive rows (from top down) indicates a single synchronous update, i.e. applying the regulatory functions on the first state gives the second state. A dark grey square indicates the ON (1) state of the node indicated below the column and white means OFF (0).

Supplementary Figure S3. The distribution of the duration of sustained ON or OFF states of each node on the asynchronous complex attractor of the Phase Switch Oscillator.

Using general asynchronous update, we sampled an extensive number of trajectories of the system on the complex attractor, where each node alternates between being ON and OFF. Each row corresponds to a node. The first figure in the row indicates the distribution of how long this node is ON (with the median in red), the second figure indicates the duration of how long this node is OFF, and the third figure indicates the distribution of the duration of a consecutive on and off period. If the complex attractor were a deterministic cycle, the duration of a consecutive on and off period for any node were 16 time steps (i.e. each of the 8 nodes turning on once and turning off once). The observed medians are very close to 16. The split between the on and off periods is even (median ON and OFF duration of 8) for five nodes and more asymmetric for three nodes, namely Cdc20, CyclinB, UbcH10. These three nodes also exhibit a slightly asymmetric pattern in the synchronous limit cycle, namely a 3 to 6 split of the nine steps as compared to the 4 to 5 split of the rest of the nodes (see Figure 5).

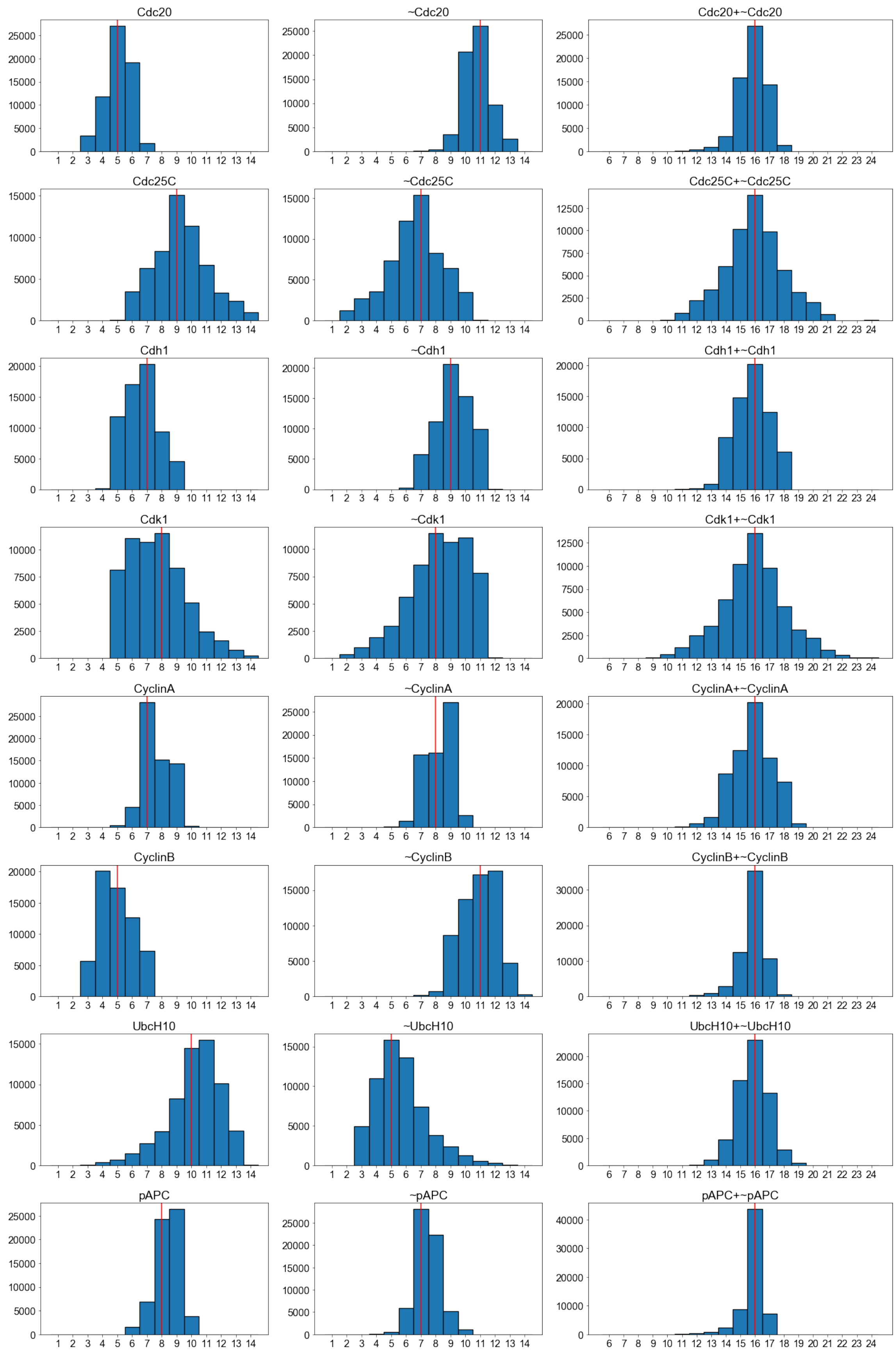
Cdc20
~Cdc20
Cdc20+~Cdc20
Cdc25C
~Cdc25C
Cdc25C+~Cdc25C
Cdh1
~Cdh1
Cdh1+~Cdh1
Cdk1
~Cdk1
Cdk1+~Cdk1
CyclinA
~CyclinA
CyclinA+~CyclinA
CyclinB
~CyclinB
CyclinB+~CyclinB
UbcH10
~UbcH10
UbcH10+~UbcH10
pAPC
~pAPC
pAPC+~pAPC

Supplementary Figure S4: Two complementary views of the relationships among conditionally stable motifs of the Phase Switch Oscillator. The information content of this figure is the same as that of Figure 8; by showing two additional views we aim to better communicate the information. In the top panel each black rectangle represents a group of conditionally stable motifs (CSMs), each of which is shown as a red rectangle. The overlaps between red rectangles illustrates that the CSMs share virtual nodes. The CSMs also activate each other, indicated as arrows between red rectangles. In the bottom panel the same groups of CSMs are characterized by the virtual nodes that participate in each. It is thus apparent that the complementary nodes of a group also form a group. Edges among groups of CSMs are defined based on the logic implication between them. For example, the virtual nodes of Cyc are sufficient to activate the virtual nodes of Cyclosome, as can be seen from the last two rows of Figure 8. The virtual nodes of ~Cyclosome together with ~UbcH10 are sufficient to activate Cyc, as shown in the second and third rows of Figure 8. Collapsing each black rectangle into a single meta-node makes the two panels identical, and also identical to the middle panel of Figure 9.

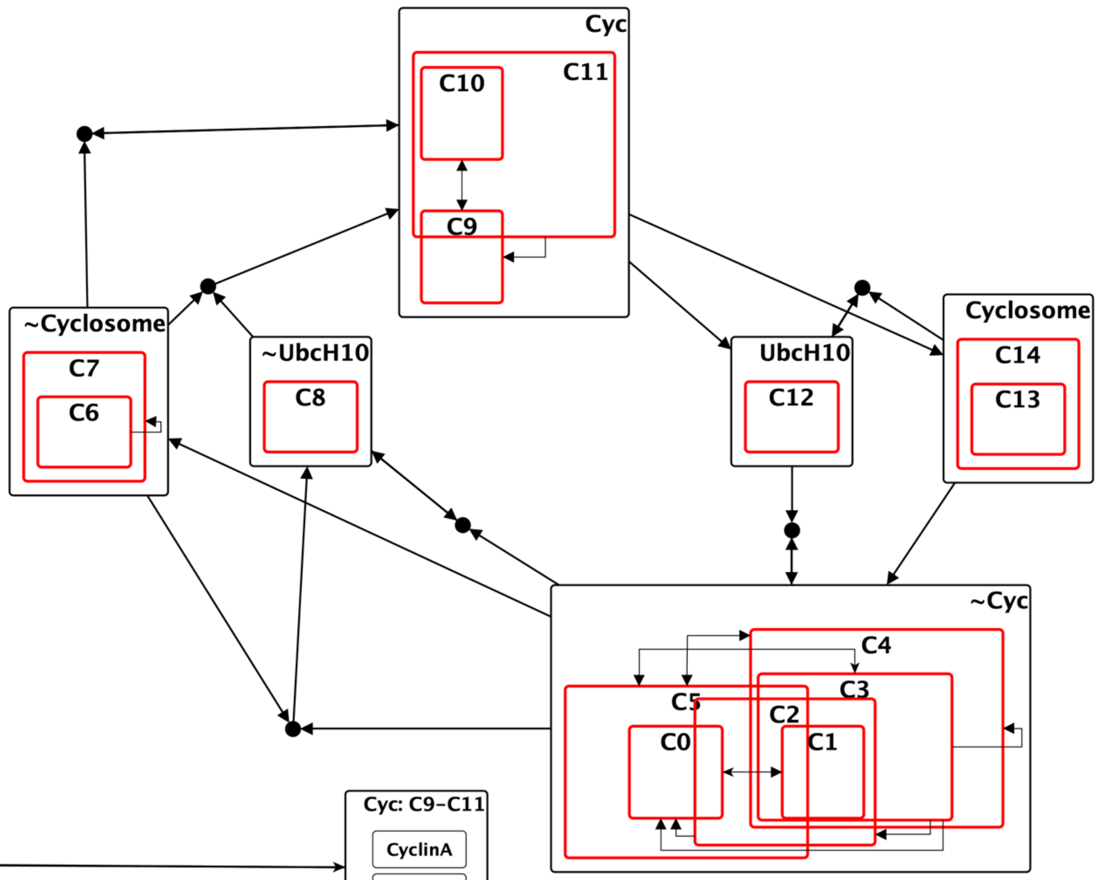
Cyc
C11
C10
C9
~Cyclosome
C7
C6
~UbcH10
C8
UbcH10
C12
Cyclosome
C14
C13
~Cyc
C4
C3
C5
C2
C0
C1

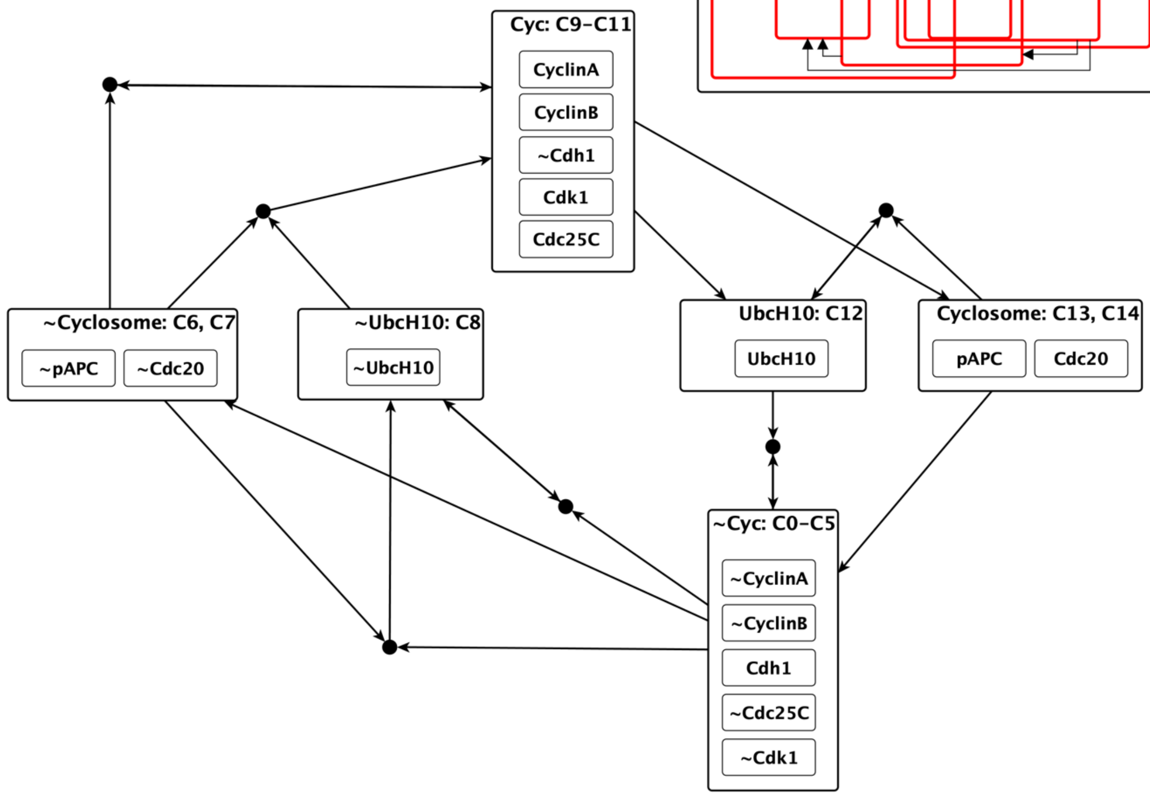
Cyc: C9–C11
CyclinA
CyclinB
~Cdh1
Cdk1
Cdc25C
~Cyclosome: C6, C7
~pAPC
~Cdc20
~UbcH10: C8
~UbcH10
UbcH10: C12
UbcH10
Cyclosome: C13, C14
pAPC
Cdc20
~Cyc: C0–C5
~CyclinA
~CyclinB
Cdh1
~Cdc25C
~Cdk1

Supplementary Figure S5. A sequence of sustained states of CyclinB can induce a sequence of transitions between attractors that mimics the cell cycle progression.

The results are the average of 1000 simulations using general asynchronous update with random initial conditions. The unit of time is update of a single node (which may or may not result in a state change). The state of the system is characterized by the overlap with the three attractors of the Phase Switch, where the maximal overlap is seven (the value of CyclinB is not included in calculating the overlap). In this sequence, CyclinB is manipulated to drive the system ensemble from random initial states through two cycles of the phase sequence G2 $\rightarrow$ SAC $\rightarrow$ G0/G1. A grey background indicates that CyclinB is forced off, while a white background indicates that it is forced on. Locking CyclinB off yields a G2-like state in all the trajectories; this can be seen from the fact that the average overlap with the G2 attractor stabilizes at six after about 75 steps. The difference of this state from the G2 attractor is in the state of Cdk1, which is ON in this state and OFF in the G2 attractor (see Supplementary Table S1). When CyclinB is subsequently forced on (t=150), all trajectories converge into an SAC-like state, which differs from the SAC attractor in that Cdc20 is ON. At the next time CyclinB is kept off (t=300), the trajectories transiently approach the G0/G1 attractor (indicated by the blue peak), but eventually converge to the G2 state. Keeping CyclinB on again (t=450) yields the SAC state. If CyclinB is subsequently off for a short duration only (t=600 to t=610), the system can converge to the G0/G1 attractor in the majority of trajectories. This is because the SAC-like state has CyclinA = 0, which, together with CyclinB = 0, is sufficient for Cdh1 = 1, Cdc25c = 0, and Cdk1 = 0. Furthermore, Cdh1 = 1 is sufficient for Cdc20 = 0, which is itself sufficient for pAPC = 0, thus at this point all nodes are OFF, as in the G0/G1 attractor. If CyclinB is turned on before Cdc25c and Cdk1 deactivate, the system will return to the SAC-like state. Conversely, if CyclinB is held off for sufficiently long, the system will pass through a G0/G1-like state, but the OFF state of Cdc20 and pAPC (i.e. the OFF state of the Cyclosome metanode, see Figure 9) is sufficient to turn CyclinA ON, and the system ultimately converges to the G2-like attractor (as in the interval from t=300 to t=450). Thus, there exists a narrow window of CyclinB deactivation duration that causes convergence to the G0/G1-like state. The ideal duration for convergence to this state depends on the update order. In our simulations, a duration of ten time steps causes the greatest number of update orders to converge to the G0/G1-like state, and with CyclinB inactivation durations less than five or more than fifteen time steps, a negligible fraction of trajectories settle in the G0/G1-like state.

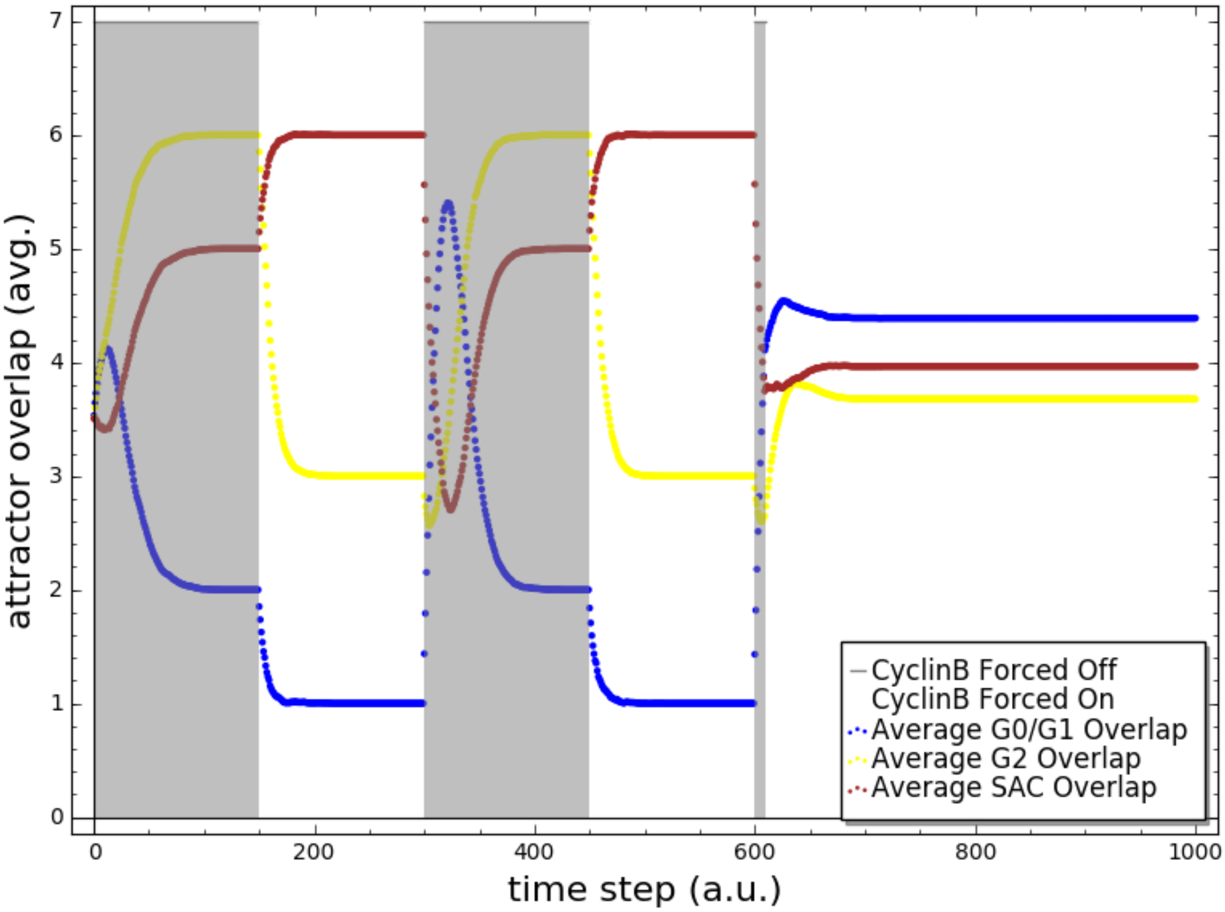
attractor overlap (avg.)
time step (a.u.)
CyclinB Forced Off
CyclinB Forced On
Average G0/G1 Overlap
Average G2 Overlap
Average SAC Overlap

Supplementary Figure S6. The cycle graph constructed for the Phase Switch Oscillator. Each node of this graph represents a positive feedback loop of the regulatory network with node states (top row of each node label) that become self-sustaining when the state of certain other nodes (bottom row of each node label) is held fixed. As in previous figures the OFF state of a node is represented by a ~ preceding the respective node name. An undirected edge between nodes of the cycle graph indicates that the nodes states of the two cycles and their associated conditions are mutually compatible and non-disjoint. Every node and every consistent connected subgraph of the cycle graph corresponds to a conditionally stable motif. Note that in this case, all connected subgraphs are consistent. The largest connected subgraphs correspond to the meta-nodes Cyc (a subgraph of 10 cycles in the top left), ~Cyc (an 8-cycle subgraph in the top right), ~pAPC/Cdc20 (a four-clique in the bottom right), UbcH10 (triangle in the bottom left), pAPC/Cdc20 (two cycles connected by an edge) and ~UbcH10 (single cycle).

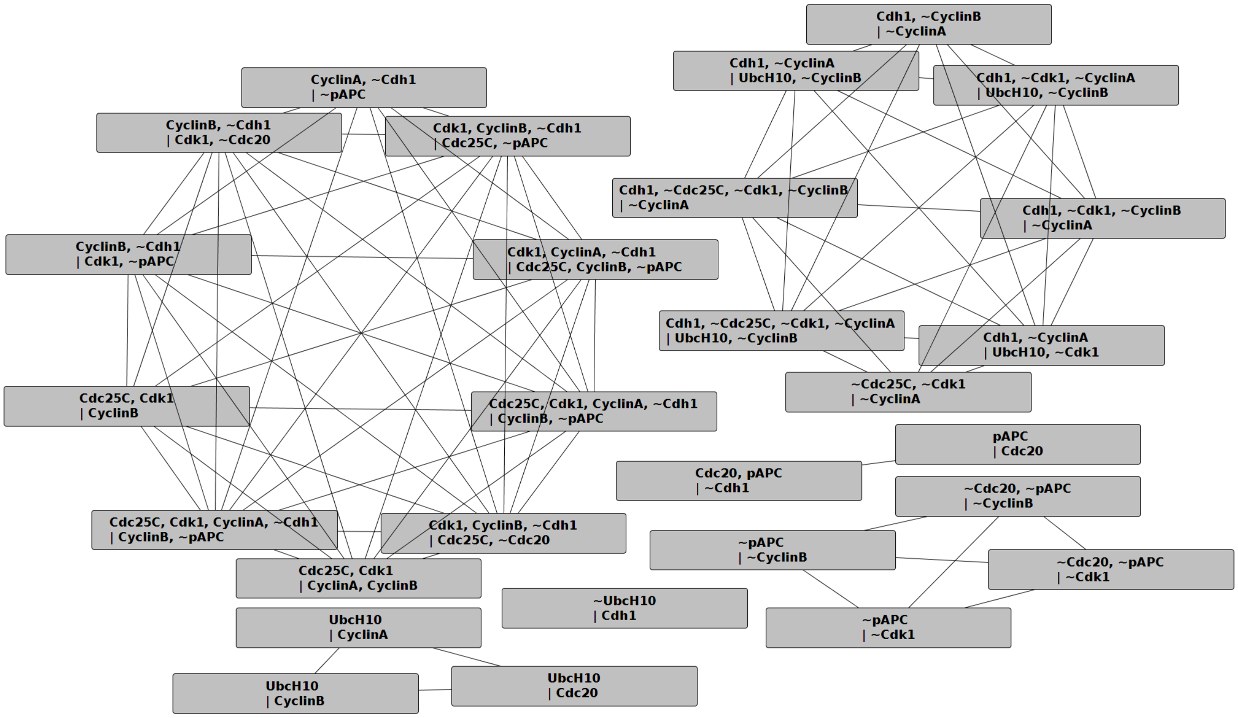

Supplement: Supplementary file 2 — Supplementary Figures [file 41598_2019_52725_MOESM2_ESM.pdf]
