# Supplementary material for: A feedback loop of conditionally stable circuits drives the cell cycle from checkpoint to checkpoint
Source: Sci Rep. 2019 Nov 11;9:16430. doi: 10.1038/s41598-019-52725-1 (PMC6848090; doi:10.1038/s41598-019-52725-1)
Supplement: Supplementary file 3 — Supplementary Notes 1-9 [file 41598_2019_52725_MOESM3_ESM.pdf]

Supplementary Notes for

A feedback loop of conditionally stable circuits drives the cell cycle from checkpoint to checkpoint

Dávid Deritei[1,2], Jordan Rozum[1], Erzsébet Ravasz Regan[3], Réka Albert[1]

[1]Department of Physics, Pennsylvania State University, University Park, PA, United States of America
[2] Department of Network and Data Science, Central European University, Budapest, Hungary
[3] Biochemistry and Molecular Biology, The College of Wooster, Wooster, OH, United States of America

Note on references: if a citation was also used in the main text, we indicate the number in the main text. Citations that appear in the Supplementary Materials only are numbered starting with S1.

Supplementary Note S1. Detailed description of the molecular processes modeled in Deritei et al. 2016

The Boolean cell cycle model by Deritei et al is composed of two semi-autonomous modules. The first module, termed the ***Restriction Switch***, models the irreversible switch cells undergo when they pass the restriction point and commit to a cell cycle irrespective of further growth stimulation. The second module, termed the ***Phase Switch***, models the switch-like transitions from G2 into mitosis, as well as spindle assembly checkpoint passage from metaphase to anaphase. The two modules are coupled to each other, as well as to the main processes driven by the cell cycle control machinery; namely DNA Replication and assembly of the mitotic spindle. Cell cycle progression in this model is dependent on a single Growth Factor input (GF). GF represents an extracellular environment with saturating mitogen levels, leading to full activation of growth signaling pathways that activate the transcription factors and G1 cyclins responsible for starting the cell cycle. Thus, when GF is on the model settles into a limit cycle and mimics continuously cycling cells. In contrast, GF = off leads to a stable state that corresponds to quiescent G0 cells.

The Restriction Switch. The mammalian restriction point marks a commitment in late G1, prior to which cells require ongoing mitogenic stimulation in order to initiate the cell cycle. Past the restriction point, division proceeds regardless of the presence of growth signals. In cells entering the cell cycle from quiescence (G0 state), growth factors stimulate the activation of Myc, a transcription factor responsible for early G1 induction of Cyclin D1, as well as its partner kinase Cdk 4 (the active complex is modeled by CyclinD1 = ON). As active Cyclin D1 / Cdk complexes accumulate, they block the activity of the cyclin dependent kinase inhibitor $p27^{Kip1}$ and phosphorylate the

retinoblastoma protein RB. This blocks RB's ability to inhibit E2F1-mediated transcription. E2F1 then orchestrates the production of a wide array of proteins required for cell cycle progression and DNA replication, including Cyclin E. As Cyclin E / Cdk2 complexes are also potent inhibitors of RB, the presence of Cyclin E guarantees that E2F1 remains active, keeping Cyclin E levels high regardless of mitogenic stimulation. At this point cells are committed to replication. In this respect, the Restriction Switch is similar to previously published models of cell cycle commitment [43, S1–S3]. In addition, the switch also includes experimentally documented feedback from E2F1 to Cyclin D1 and Myc, which allow the Restriction Switch to switch into its committed state early in metaphase (following Cyclin A degradation in pro-metaphase) and drive an additional cell cycle in the absence of growth factors.

The Phase Switch. Once cells finish DNA replication and repair all DNA damage, they undergo another irreversible switch-like transition that commits them to mitosis. Mechanistically, this commitment involves full activation of M-phase Cyclin B / Cdk1 complexes. The process starts with Cyclin B accumulation in G2, when the activity of the APC/C -- Cdh1 ubiquitin ligase complex is blocked by Cyclin A / Cdk2 activity. Once the checkpoint proteins sensing ongoing DNA replication and/or DNA damage turn OFF and Wee1 activity decreases, the inhibition of Cdk1 kinase by Wee1 is lifted. Once active, Cyclin B / Cdk1 can keep Wee1 suppressed even if DNA damage signals reappear [S4], making the commitment to Cyclin B / Cdk1 a switch-like transition. Cyclin B / Cdk1 activity initiates the processes of mitosis, including chromosome condensation, dissolution of the nuclear membrane and assembly of the mitotic spindle. In the model this complex choreography of events is simplified into the Metaphase node outside of the Phase Switch. The Metaphase node is activated by Cyclin B and Cdk1 in cells with 4N DNA content; its ON state represents the completion of spindle assembly. As long as Metaphase is OFF, the Phase Switch maintains a robust Cyclin B and Cdk1-high state where the anaphase promoting complex is phosphorylated but inactive -- the spindle assembly checkpoint. Its passage requires Metaphase = ON (a signal external to the Phase Switch) and the subsequent inhibition of Mad2. Mad2 is a highly sensitive signaling molecule that remains active as long as even a single kinetochore (and thus chromosome) remains unattached to the mitotic spindle. Once Mad2 is inactive, the Cdc20-bound and phosphorylated APC/C complex, modeled as pAPC = ON and Cdc20 = ON, degrades Cyclin B and thus inactivates mitotic cyclin / cyclin-dependent kinase activity. In addition, the complex tags the protein securin for degradation, releasing a protease (separase) that cleaves the cohesin rings that keep sister chromatids together. This marks the start of anaphase, when sister chromatids begin their movement to opposite poles of the mitotic spindle. Activity of the Cdc20-bound and phosphorylated APC/C complex is short-lived, however, as APC/C loses its Cdk1/Cyclin B-mediated phosphorylation and Cdh1 replaces Cdc20 in the complex. This leads to Cdc20 degradation, following which the Phase Switch locks into a stable state that matches that of G0/G1 cells. At this point, further external input from a committed Restriction Switch as well as the Replication node is required to toggle it back into G2 (Figure 1, right) [S5].

Checkpoints. Healthy mammalian cells pass a series of checkpoints as they execute the cell cycle, starting with checks for sufficient mitogenic stimulation [S5], adequate nutrient, energy and amino acid levels [S6], cell size [S7], and lack of DNA damage [S8] before they initiate DNA synthesis. All these checkpoints operate upstream of the Restriction Switch, in that they modulate the growth signaling pathways leading to the activation of Myc and Cyclin D. First, sustained mitogenic signals are required to keep the MAPK, PI3K / AKT1 and mTORC pathways active long enough to push the Restriction Switch into its committed state. In the absence of sufficient energy, nutrients or amino acids, this signal is interrupted by the inhibition of mTORC1, effectively vetoing the effect of proliferative signals [S6]. The effect of DNA damage on the Restriction Switch is more direct, as it activates checkpoint proteins as well as the cyclin dependent kinase inhibitor p21 to block all Cyclin / cyclin dependent kinase activity. The molecular mechanism by which cell size is monitored and linked to the restriction point is not well understood (though the existence of this checkpoint is well documented), but it appears to delay restriction point passage in small cells [S7]. The Deritei 2016 model does not include a mechanism for any of these requirements except for mitogenic stimulation, and assumes that all other G1 checkpoints act to block the signal from the input, GF, to the rest of the network, effectively turning it off. (The followup model Sizek et al 2019 [19] explicitly accounts for the main growth signaling pathways that link growth receptors to the Restriction Switch, but does not focus on checkpoint control of these pathways.) Once in G2, cells need to clear a DNA damage checkpoint to start mitosis. This checkpoint operates by maintaining inhibitory Cdk1 phosphorylation, a point of convergence for signals that block mitotic entry. In the model, this checkpoint is operational in the presence of ongoing DNA replication (which activates some of the same pathways as single-strand DNA damage, included in [19]), but the model does not explicitly induce DNA damage sensing signaling [19, S9]. Finally, cells must pass the spindle assembly checkpoint to advance from metaphase to anaphase. The model contains one key mediator of this checkpoint, Mad2, but it does not explicitly include ways to simulate failed spindle assembly beyond artificial inhibition of the Metaphase node. Thus, the model is well suited for the study of optimal cell cycle progression where passage through most checkpoints occurs without delay. In addition, the model incorporates the main "entry points" through which different checkpoints can stop the cycle in G1, G2 or at SAC.

In the following two tables we indicate the full names of the nodes included in the Deritei et al. 2016 model, and the interactions within the Phase Switch.

Table S1. 1. Descriptions of the nodes of the Deritei et al 2016 cell cycle model

CyclinD1	active complex of Cyclin D1 and cyclin-dependent kinases 4 or 6
CyclinE	active complex of Cyclin E and cyclin-dependent kinase 2
E2F1	E2F family transcription factor 1
Myc	Myc transcription factor
p27Kip1	cyclin-dependent kinase inhibitor 1B
RB	hypho-phosphorylated Retinoblastoma protein
Cdc20	cell-division cycle protein 20

Cdc25A	cell-division cycle protein 25A
Cdc25C	cell-division cycle protein 25C
Cdh1	complex of Cdh1 and anaphase-promoting complex
Cdk1	cyclin-dependent kinase 1
CyclinA	Cyclin A
CyclinB	Cyclin B
Mad2	mitotic arrest deficient 2
pAPC	phosphorylated anaphase-promoting complex
UbcH10	ubiquitin conjugating enzyme UbcH10
Wee1	nuclear kinase Wee1
Replication	DNA replication, turns OFF when cells double their DNA content.
Metaphase	The Metaphase abstract node turns ON when spindle assembly is completed, and none of the kinetochores are left unattached.
4N_DNA	The 4N_DNA abstract node represents the complete duplication of a cell's DNA.
GF	Growth factors in the cell's external environment

Table S1.2. Interactions of the Phase Switch

Regulator	Action	Target	Reference
Cdh1	inhibits	Cdc20	[S10], [S11]
Mad2	inhibits	Cdc20	[S12-S14]
pAPC	activates	Cdc20	[S10], [S15]
Cdh1	inhibits	Cdc25A	[S10], [S16]
CyclinA	activates	Cdc25A	[S17], [S18]
CyclinA	activates	Cdc25C	[S19]
CyclinB	activates	Cdc25C	[S19], [S20]
Cdk1	activates	Cdc25C	[S19], [S20]
CyclinA	inhibits	Cdh1	[S10], [S21]
CyclinB	inhibits	Cdh1	[S10], [S21]
Cdk1	inhibits	Cdh1	[S10], [23]
Cdc25C	activates	Cdk1	[S19], [S20], [S22]
CyclinA	activates	Cdk1	[S22]
CyclinB	activates	Cdk1	[S22]
Wee1	inhibits	Cdk1	[S23]
Cdk1	activates	Cdk1	[S24]
Cdc25A	activates	CyclinA	[S25], [S26]
CyclinA	activates	CyclinA	
pAPC	inhibits	CyclinA	[S27]
Cdc20	inhibits	CyclinA	[S27]

Cdh1	inhibits	CyclinA	[S21], [12], [S28]
UbcH10	inhibits	CyclinA	[S28]
pAPC	inhibits	CyclinB	[S21]
Cdc20	inhibits	CyclinB	[S21]
Cdh1	inhibits	CyclinB	[S21]
pAPC	inhibits	Mad2	[S13]
Cdc20	inhibits	Mad2	[S13]
CyclinB	activates	Mad2	[S29], [S30]
Cdk1	activates	Mad2	[S29], [S30]
pAPC	activates	pAPC	[S12], [S31]
Cdc20	activates	pAPC	[S12]
CyclinB	activates	pAPC	[S12], [S32]
Cdk1	activates	pAPC	[S12], [S32]
Cdh1	inhibits	UbcH10	[S28]
Cdc20	activates	UbcH10	[S28]
CyclinA	activates	UbcH10	[S28]
CyclinB	activates	UbcH10	[S28]
UbcH10	activates	UbcH10	[S28]
Cdk1	inhibits	Wee1	[S24]
CyclinA	inhibits	Wee1	[S24]
CyclinB	inhibits	Wee1	[S24]
Interactions incident on the Phase Switch			
E2F1	activates	Cdc25A	[S33]
CyclinE	activates	Cdc25A	[S17]
E2F1	activates	CyclinA	[S34]
Replication	activates	Wee1	[S35]
Metaphase	inhibits	Mad2	[S14]
4N_DNA	activates	Mad2	[S14]

Supplementary Table S1.3. States of the cyclic attractor of the synchronous cell cycle model of Deritei et al. This limit cycle is obtained in the sustained presence of growth factors. The rows of the table represent the 13 states of the limit cycle, while the columns denoted by node names represent the states of individual nodes in each state. We group the states according to the cell cycle phases, indicated in the leftmost column. The state labels correspond to the labels shown in the top right panel of Figure 1. We group the nodes into three categories, namely the Phase Switch, the Restriction Switch and three abstract nodes. We highlight the states of the Restriction Switch or Phase Switch that are closest to a respective attractor. The two attractors of the Restriction Switch are E2F1=CyclinE=CyclinD1=Myc=1, RB=p27Kip1=0 (denoted Past Restriction, and marked in pink in Figure 1) and E2F1=CyclinE=CyclinD1=Myc=0, RB=p27Kip1=1 (denoted Before Restriction and marked in bright green). The three

attractors of the Phase Switch are indicated in Supplementary Table S1. The color code of the module attractors is the same as in Figure 1.

Phase	State label	Restriction Switch						Phase Switch											abstract nodes		
		E2F1	CyclinE	CyclinD1	RB	p27Kip1	Myc	CyclinA	Cdc25C	Cdc25A	CyclinB	Cdh1	Cdk1	Wee1	UbcH10	pAPC	Cdc20	Mad2	Replication	4N DNA	Metaphase
G1	CC1	1	1	1	0	0	1	0	0	1	0	1	0	0	1	1	0	0	0	0	0
S	CC2	1	1	1	0	0	1	0	0	1	0	1	0	0	0	0	0	0	1	0	0
	CC3	1	1	0	0	0	1	1	0	1	0	1	0	1	0	0	0	0	1	0	0
	CC4	0	1	0	0	0	1	1	1	1	0	0	0	1	0	0	0	0	1	0	0
G2	CC5	0	0	0	0	0	1	1	1	1	1	0	0	1	1	0	0	0	1	1	0
	CC6	0	0	0	0	1	1	1	1	0	1	0	0	1	1	0	0	0	0	1	0
	CC7	0	0	1	1	1	1	1	1	0	1	0	0	0	1	0	0	0	0	1	0
M	CC8	0	0	1	0	0	1	1	1	0	1	0	1	0	1	0	0	0	0	1	0
	CC9	0	0	1	0	0	1	1	1	0	1	0	1	0	1	0	0	1	0	1	1
	CC10	0	0	1	0	0	1	0	1	0	1	0	1	0	1	1	0	0	0	1	1
	CC11	1	0	1	0	0	1	0	1	0	1	0	1	0	1	1	1	0	0	1	1
C	CC12	1	0	1	0	0	1	0	1	0	0	0	1	0	1	1	1	0	0	1	0
	CC13	1	1	1	0	0	1	0	0	0	0	1	0	0	1	1	1	0	0	1	0
G1	CC1	1	1	1	0	0	1	0	0	1	0	1	0	0	1	1	0	0	0	0	0

Supplementary Note S2. Isolating the Phase Switch Module from the Boolean model of the cell cycle

Generally speaking, isolating a designated subset of a network's nodes means that only the edges that start and end at nodes of this subset are kept and the rest are deleted. For the dynamical system, the most important implication of this process is that certain influences incident on the nodes of the subset will be disregarded. In the specific case of a Boolean dynamical system in each of these nodes' regulatory functions only the terms that represent regulation from within the group are kept; the regulation that comes from outside of the group is disregarded. This isolation is a valid reflection of the original, full system if certain conditions about the disregarded regulators are satisfied. Otherwise, it is a useful approximation.

Consider a hypothetical node X within the designated subset, which has two regulators, one inside the designated subset (denoted Int) and one outside of it (denoted Ext). The regulatory function of X takes one of four possibilities, indicated in Table S2.1. The regulatory function of X after isolation of the designated subset will be "f_X = Int ". This is consistent with the original regulatory function only if the external regulator Ext is in a fixed state, specified in the second column of Table S2.1. The

consistency is due to the property of Boolean functions, e.g. "A or 0 = A", "A and 1 = A". If the condition is not satisfied, the external regulator overrides the internal regulator and determines the outcome of the regulatory function of X (see the third column of Table S2.1).

Table S2.1

Regulatory function of X in the original system	Condition on Ext for which the "f_X= Int" approximation is valid	Outcome of the regulatory function of X if the condition is not satisfied
f_X= Int or Ext	Ext = 0	f_X= 1
f_X = Int and Ext	Ext = 1	f_X= 0
f_X = Int or not Ext	Ext = 1	f_X= 0
f_X = Int and not Ext	Ext = 1	f_X= 1

A notable special case of the general situation described above is when the internal regulation contains stable motifs and thus allows multi-stability. To illustrate this case, we consider the simple case wherein the system consists of X, which activates itself, and an external regulator Ext. The subsystem f_X= X is bistable. This bistability is preserved for one of the values of Ext (second column of Table S2.2). The other value of Ext selects one of the fixed points of X.

Table S2.2

Regulatory function of X in the original system	Condition on Ext for which the bistability of X is preserved	Outcome of the regulatory function of X if the condition is not satisfied
f_X= X or Ext	Ext = 0	f_X= 1
f_X = X and Ext	Ext = 1	f_X= 0
f_X = X or not Ext	Ext = 1	f_X= 0
f_X = X and not Ext	Ext = 1	f_X= 1

In general, certain values of the external regulator(s) allow the full attractor repertoire of the isolated subsystem and other values reduce the attractor repertoire.

When isolating a subset of a Boolean system by severing the external edges incident on this system, one needs to find the states of external inputs (i.e. starting nodes of the incident edges) that best preserve the dynamical influence of the internal nodes. Deritei et al. [17] propose to use a combination of frozen inputs such that a) all remaining input nodes are functional (i.e., they are able to impact the output in some way), and b) the entropy of the remaining Boolean regulatory function is as large as possible. These input combinations are chosen independently for each node of the subsystem. Contradictory external conditions (e.g., external node A needs to be 0 in order to not override internal node X, but 1 in order to not override internal node Y) are allowed. Thus, a module embedded in a larger network may have its autonomy partially impeded at different times, depending on the rest of the network.

We now apply this analysis to the Phase Switch module. The Phase Switch module of the cell cycle model was defined by Deritei et al. as being made up of 11 nodes: CyclinA, CyclinB, Cdk1, Cdh1, Cdc25A, Cdc25C, Cdc20, Mad2, pAPC, UbcH10,

Wee1. Four of these nodes receive inputs from nodes other than these 11: CyclinA and Cdc25A are regulated by two nodes of the Restriction Switch, and Mad2 and Wee1 receive inputs from the three abstract nodes (see Figure 1). Specifically, CyclinA is regulated by E2F1, Cdc25A is regulated by E2F1 and CyclinE, Wee1 is regulated by the abstract node Replication, and Mad2 receives inputs from the abstract nodes Metaphase and 4N DNA. The first group of regulatory functions of Supplementary Text S3 indicate the regulatory functions of the 11 nodes of the Phase Switch in the cell cycle model, and the second group of regulatory functions indicates the regulatory functions that only consider the regulation internal to the Phase Switch. Comparing the regulatory functions of the four nodes that do receive external inputs, we can see that the conditions for the validity of the module-autonomous regulatory functions are:

- CyclinA node: E2F1=1
- Cdc25A node: E2F1 =1 or CyclinE=1 but not both
- Wee1 node: Replication = 1
- Mad2 node: Metaphase=0 and 4N DNA = 1

The union of all of these conditions, i.e., E2F1=1, CyclinE=0, Replication = 1, Metaphase=0, 4N DNA=1, can only be satisfied transiently. This is because DNA replication is the process that generates 4N DNA (doubled DNA content) and stops after cells double their DNA content (see Supplementary Table S3 of [17]); thus the simultaneous ON state of Replication and 4N DNA is short-lived. The impossibility of satisfying the union of the conditions means that there is no sustained period during the cell cycle during which the Phase Switch is truly autonomous.

While the Phase Switch module is never truly isolated from the rest of the cell cycle network, the properties of the artificially isolated Phase Switch are still informative. Specifically, cell states that satisfy different subsets of the above condition select from the attractor repertoire of the isolated Phase Switch.

1. E2F1=1, CyclinE=0, Replication = 0, Metaphase=0, 4N DNA=1

Replication = 0 causes that f_{Wee1} = 0, thus Wee1 will stabilize at 0. This eliminates the P1 motif, and eliminates the fourth and fifth paths in the stable motif succession diagram of Figure 3. However, the P0 and P2 motifs and the rest of the paths are intact. The Phase Switch can converge into the SAC or G0/G1 attractors, thus it can describe the transition past the spindle assembly checkpoint and into cytokinesis. Note that the state of E2F1 and CyclinE do not coincide with a point attractor of the Restriction Switch.

2. E2F1=1, CyclinE=1, Replication = 1, Metaphase=0, 4N DNA=1

The coincident activity of E2F1 and CyclinE causes that f_{Cdc25A}= 1, thus Cdc25A will stabilize at 1. This eliminates the P0 motif and the five paths of the stable motif succession diagram that contain it. Nevertheless, the P1 and P2 motifs and two paths are intact, thus this system can describe the exit from mitosis.

3. E2F1=1, CyclinE=1, Replication = 0, Metaphase=1

The Metaphase condition (which marks the completion of spindle assembly) causes f_{Mad2}= 0, which leads to the elimination of the P2 motif and thus of the SAC attractor.

The coincident activity of E2F1 and CyclinE causes that f_{Cdc25A}= 1, thus Cdc25A will stabilize at 1. This eliminates the P0 motif. Replication = 0 causes that f_{Wee1} = 0, thus Wee1 will stabilize at 0. This eliminates the P1 motif, and eliminates the fourth and fifth paths in the stable motif succession diagram. Thus, all of the attractors of the Phase Switch are eliminated. This condition represents the Phase Switch Oscillator.

Supplementary Note S3. Regulatory functions of the Phase Switch module and the Phase Switch Oscillator

Regulatory functions of the nodes of the Phase Switch module in the full cell cycle model

Regulators that are outside of the Phase Switch module are indicated in bold.

f_{Cdc25A}= (**E2F1** and **CyclinE**) or (**E2F1** and CyclinA) or (CyclinA and not Cdh1 and **CyclinE**)
f_{Mad2}= (**4N_DNA** and not pAPC and CyclinB and Cdk1 and not **Metaphase**) or (**4N_DNA** and not Cdc20 and CyclinB and Cdk1 and not **Metaphase**)
f_{Wee1}= (not CyclinA and not CyclinB and **Replication**) or (not Cdk1 and **Replication**)
f_{Cdc25C}= CyclinA or (CyclinB and Cdk1)
f_{Cdh1}= (not CyclinA and not CyclinB) or (not CyclinA and not Cdk1)
f_{Cdk1}= (Cdc25C and CyclinA and Cdk1) or (Cdc25C and CyclinB and Cdk1) or (Cdc25C and CyclinA and not Wee1) or (Cdc25C and CyclinB and not Wee1)
$f_{CyclinA}$= (not pAPC and not Cdh1 and Cdc25A and **E2F1**) or (not pAPC and not UbcH10 and Cdc25A and **E2F1**) or (not Cdc20 and not Cdh1 and Cdc25A and **E2F1)** or (not Cdc20 and not UbcH10 and Cdc25A and **E2F1)** or (not pAPC and not Cdh1 and CyclinA) or (not pAPC and not UbcH10 and CyclinA) or (not Cdc20 and not Cdk1 and CyclinA) or (not Cdc20 and not UbcH10 and CyclinA)
$f_{CyclinB}$= (not pAPC and not Cdh1) or (not Cdc20 and not Cdh1)
f_{pAPC}= (pAPC and Cdc20) or (CyclinB and Cdk1)
f_{Cdc20}= pAPC and not Cdh1 and not Mad2
f_{UbcH10}= not Cdh1 or (UbcH10 and Cdc20) or (UbcH10 and CyclinA) or (UbcH10 and CyclinB)

Regulatory functions of the Phase Switch module

f_{Cdc25A}=CyclinA and not Cdh1
f_{Mad2}= (not pAPC and CyclinB and Cdk1) or (not Cdc20 and CyclinB and Cdk1)
f_{Wee1}= (not CyclinA and not CyclinB) or not Cdk1
f_{Cdc25C}= CyclinA or (CyclinB and Cdk1)
f_{Cdh1}= (not CyclinA and not CyclinB) or (not CyclinA and not Cdk1)
f_{Cdk1}= (Cdc25C and CyclinA and Cdk1) or (Cdc25C and CyclinB and Cdk1) or (Cdc25C and CyclinA and not Wee1) or (Cdc25C and CyclinB and not Wee1)
$f_{CyclinA}$= (not pAPC and not Cdh1 and Cdc25A) or (not pAPC and not UbcH10 and Cdc25A) or (not Cdc20 and not Cdh1 and Cdc25A) or (not Cdc20 and not UbcH10 and Cdc25A) or (not pAPC and not Cdh1 and CyclinA) or (not pAPC and not UbcH10 and

CyclinA) or (not Cdc20 and not Cdk1 and CyclinA) or (not Cdc20 and not UbcH10 and CyclinA)
$f_{CyclinB}$= (not pAPC and not Cdh1) or (not Cdc20 and not Cdh1)
f_{pAPC}= (pAPC and Cdc20) or (CyclinB and Cdk1)
f_{Cdc20}= pAPC and not Cdh1 and not Mad2
f_{UbcH10}= not Cdh1 or (UbcH10 and Cdc20) or (UbcH10 and CyclinA) or (UbcH10 and CyclinB)

Regulatory functions of the Phase Switch Oscillator (after substituting Cdc25A=1, Mad2=Wee1=0)
f_{Cdc25C}= CyclinA or (CyclinB and Cdk1)
f_{Cdh1}= (not CyclinA and not CyclinB) or (not CyclinA and not Cdk1)
f_{Cdk1}= (Cdc25C and CyclinA) or (Cdc25C and CyclinB)
$f_{CyclinA}$= (not pAPC and not Cdh1) or (not pAPC and not UbcH10)
$f_{CyclinB}$= (not pAPC and not Cdh1) or (not Cdc20 and not Cdh1)
f_{pAPC}= (pAPC and Cdc20) or (CyclinB and Cdk1)
f_{Cdc20}= pAPC and not Cdh1
f_{UbcH10}= not Cdh1 or (UbcH10 and Cdc20) or (UbcH10 and CyclinA) or (UbcH10 and CyclinB)

Regulatory functions of the Phase Switch Oscillator embodied in the expanded network
f_{Cdc25C}= CyclinA or (CyclinB and Cdk1)
$f_{\sim Cdc25C}$= (~CyclinA and ~CyclinB) or (~CyclinA and Cdk1)
f_{Cdh1}= (~CyclinA and ~CyclinB) or (~CyclinA and ~Cdk1)
$f_{\sim Cdh1}$= CyclinA or (CyclinB and Cdk1)
f_{Cdk1}= (Cdc25C and CyclinA) or (Cdc25C and CyclinB)
$f_{\sim Cdk1}$= ~Cdc25C or (~CyclinA and ~CyclinB)
$f_{CyclinA}$= (~pAPC and ~Cdh1) or (~pAPC and ~UbcH10)
$f_{\sim CyclinA}$= pAPC or (Cdh1 and UbcH10)
$f_{CyclinB}$= (~pAPC and ~Cdh1) or (~Cdc20 and ~Cdh1)
$f_{\sim CyclinB}$= (pAPC and Cdc20) or Cdh1
f_{pAPC}= (pAPC and Cdc20) or (CyclinB and Cdk1)
$f_{\sim pAPC}$= (~pAPC and ~CyclinB) or (~Cdc20 and ~CyclinB) or (~pAPC and ~Cdk1) or (~Cdc20 and ~Cdk1)
f_{Cdc20}= pAPC and ~Cdh1
$f_{\sim Cdc20}$= ~pAPC or Cdh1
f_{UbcH10}= ~Cdh1 or (UbcH10 and Cdc20) or (UbcH10 and CyclinA) or (UbcH10 and CyclinB)
$f_{\sim UbcH10}$= (Cdh1 and ~UbcH10) or (Cdh1 and ~Cdc20 and ~CyclinA and ~CyclinB)

Supplementary Note S4. Comparing complex attractors under synchronous and asynchronous update.

Here we summarize literature that addresses how a synchronous limit cycle of a Boolean model may change when switching to general asynchronous update.

Garg et al. [S39] categorize cyclic attractors of synchronous systems into two categories: simple loops, where each state transition is a change of a single node, and type-2 simple loops, where more than one node changes state in a state transition. According to this categorization the PSO's cycle is a type-2 simple loop. They categorize complex attractors of general asynchronous systems into simple loops and complex loops; the latter are made up by intersecting simple loops. In this sense, the PSO’s complex attractor is a complex loop. The paper concludes that simple loops found by synchronous update will also be present under general asynchronous update of the same network, but the existence of a type-2 simple loop under synchronous update does not have any implication to the potential existence of a related complex loop under asynchronous update.

Analyzing a directed cycle of nodes in which each node has a single regulator, the article [S40] finds that it has two fixed points and a large number of cyclic attractors under synchronous update but no complex attractors (only the two fixed points) for general asynchronous update. Next, they consider the ensemble of networks in which each node has two regulators; these networks are made up by cycles and trees (acyclic networks). Finally, they conclude "*in critical k = 2 networks with asynchronous stochastic update, the number of attractors grows as a power law in N, which is in strong contrast to the synchronous case, where the number of attractors increases like a stretched exponential function.*" Thus, the vast majority of cyclic attractors obtained for synchronous update disappear when switching to general asynchronous update.

The article [S41] compares synchronous update and three types of asynchronous update on a Boolean model of abscisic acid induced closure [S42]. The model has a fixed point and two cyclic attractors under synchronous update. The cyclic attractors are destroyed under general asynchronous update.

Fauré et. al. [12] study a 10-node model of the cell cycle, including 5 nodes of the Restriction Switch (CyclinD, CyclinE, RB, p27, E2F) and 5 nodes of the Phase Switch (CyclinA, CyclinB, Cdh1, Cdc20, UbcH10). They find a 7- state limit cycle under synchronous update and a 112-state complex attractor under general asynchronous update. The complex attractor contains the nodes of the synchronous cycle. Although the article does not report an analysis of the complex attractor, based on its visualization (top panel of Figure 2 in [12]) one can conclude that the complex attractor does not preferentially visit the states of the limit cycle. Fauré et al. propose a hybrid between synchronous and asynchronous update that yields a reduced complex attractor of 18 states, including the 7 nodes of the synchronous cycle. Even this hybrid complex attractor has a higher chance of skipping a state of the synchronous limit cycle than the PSO’s complex attractor (compare the bottom right panel of Figure 2 in [12] with Figure 5 in the main text).

In conclusion, because the PSO's synchronous cycle is a type-2 loop in Garg et al.'s categorization [S39], it cannot be preserved when switching to general asynchronous update. However, since the PSO does not have point attractors, and

every Boolean system must have at least one attractor, the PSO must have a complex attractor (or more). There are no known general principles that would govern the relationship between the synchronous limit cycle and the complex attractor. The closest previous analysis [12] did not find a strong relationship other than inclusion of the 7 nodes of the limit cycle in the complex attractor.

Supplementary Note S5. Detailed description of the complex attractor of the Phase Switch Oscillator

The technical terms used in this note are described in the section "Key concepts of Boolean modeling", in the Methods, and in the Glossary (Supporting Note S10).

In the following we describe the state transitions involved in the complex attractor of the PSO, starting with the state closest to the G0/G1 attractor of the Phase Switch. As the G0/G1 state overlaps the G2 state in three node states (see Supplementary Table S2), five nodes need to change from the close-to-G0/G1 to the close-to-G2 state: CyclinB, UbcH10, CyclinA, Cdc25C need to turn ON, Cdh1 needs to turn OFF. CyclinA turns ON first (in a transition shared with synchronous update, see Figure 5). In most trajectories this leads to Cdh1 turning off and Cdc25C turning on, in either order. Next, CyclinB and UbcH10 turn ON in either order, with the Cdk1-ON transition mixed in. A considerable fraction of the asynchronous trajectories mixes the state changes of Cdh1, Cdc25C, CyclinB, UbcH10, Cdk1, and thus form transition paths between the state with overlap triple (7,4,1) and the post-G2 state, that skip the state (5,6,3). There also is a small fraction of trajectories wherein the state change of UbcH10 is delayed; these trajectories skip the post-G2 state. The rest of the trajectories follow the two steps of the synchronous update. The state closest to the G2 attractor is contained in half of the trajectories between the state (7,4,1) and the post-G2 state.

As the state closest to G2 overlaps the state closest to SAC in five node states, only three nodes need to change state to switch from the G2 to SAC state: pAPC and Cdk1 need to turn ON, while CyclinA needs to turn OFF. Cdk1 turns on first, then pAPC (in a transition shared by synchronous update). Then CyclinA turns off and Cdc20 turns on, in either order, marking a transition between the near-SAC state to the post-SAC state. The SAC state is visited by the trajectories wherein CyclinA turns off first and then Cdc20 turns on (approximately half of the total trajectories, see Supplementary Table S3). There are also a few trajectories where the turning ON of UbcH10 is delayed, and follows the turning ON of pAPC, Cdc20, or the turning OFF of CyclinA.

Finally, six nodes need to change state from the SAC to the G0/G1 state: pAPC, Cdk1, CyclinB, UbcH10, and Cdc25C need to turn off, while Cdh1 needs to turn on. In addition, Cdc20 first turns on (during the transition from the near-SAC to the post-SAC state), then it turns off during the approach to the G0/G1 state. CyclinB turns off in a transition shared by the synchronous cycle, between the post-SAC state and the state with attractor overlap (2,3,6). It is also possible that the turning off of Cyclin B precedes that of CyclinA, creating trajectories from the near-SAC state to (2,3,6) that skip the post-SAC state. Following the state (2,3,6), the Cdh1-on, Cdc25C-off and Cdk1-off transitions can occur in variable order. Cdc20, pAPC, and UbcH10 turn off in variable order following the activation of Cdh1, in trajectories that reach the G0/G1 state with or without visiting the states (5,2,3) and (6,3,4).

Supplementary Note S6. Topological analysis of the expanded network of the Phase Switch Oscillator

The technical terms used in this description are described in the section "Key concepts of Boolean modeling", in the Methods and in the Glossary (Supporting Note S10).
As we describe in the main text and show in Figure 4, we obtain the Phase Switch Oscillator from the Phase Switch by assuming that the conditions of the three cell cycle checkpoints (the restriction point, the DNA damage checkpoint and the spindle assembly checkpoint) are satisfied. Another, practical way of doing the same is to start from the isolated Phase Switch (i.e., the second group of regulatory functions in Supplementary Note S3) and fix the states Cdc25A = 1, Wee1 = Mad2 = 0. Each of these states contradicts one of the three stable motifs of the Phase Switch. The virtual nodes corresponding to these fixed node states have out-edges only to composite nodes, thus no further node states stabilize as a direct consequence of their stabilization (see Supplementary Figure S1). Moreover, these fixed node states do not create new stable motifs. In the Phase Switch there is only one alternative combination of fixed node states that eliminates the original stable motifs and does not create new ones: Cdc25A=1, Cdk1=1, Mad2=0. This alternative combination has the same biological meaning as the one we considered.

The expanded network of the Phase Switch Oscillator contains 16 virtual nodes and 21 composite nodes (see Supplementary Figure S6.1). Its 72 edges form 31 sufficient relationships between virtual nodes, each of which is either direct or mediated by a single composite node. Each of these sufficient relationships appears as a disjunctive ("or"-separated) clause in the regulatory function of the target node. For example, as shown in the last section of Supplementary Note S3, the regulatory function of Cdc25C is f_{Cdc25C}= CyclinA or (CyclinB and Cdk1). Both terms separated by the "or" operator, i.e. "Cyclin A" and "CyclinB and Cdk1" indicate a sufficient regulatory relationship, meaning that either the activity of Cyclin A, or the simultaneous activity of CyclinB and Cdk1 can cause the activation of Cdc25C. Consequently, Cdc25C (a green-highlighted node in the top part of the expanded network) has two incoming edges, one from CyclinA and one from a composite node that in turn has two incoming edges, one from CyclinB and one from Cdk1.

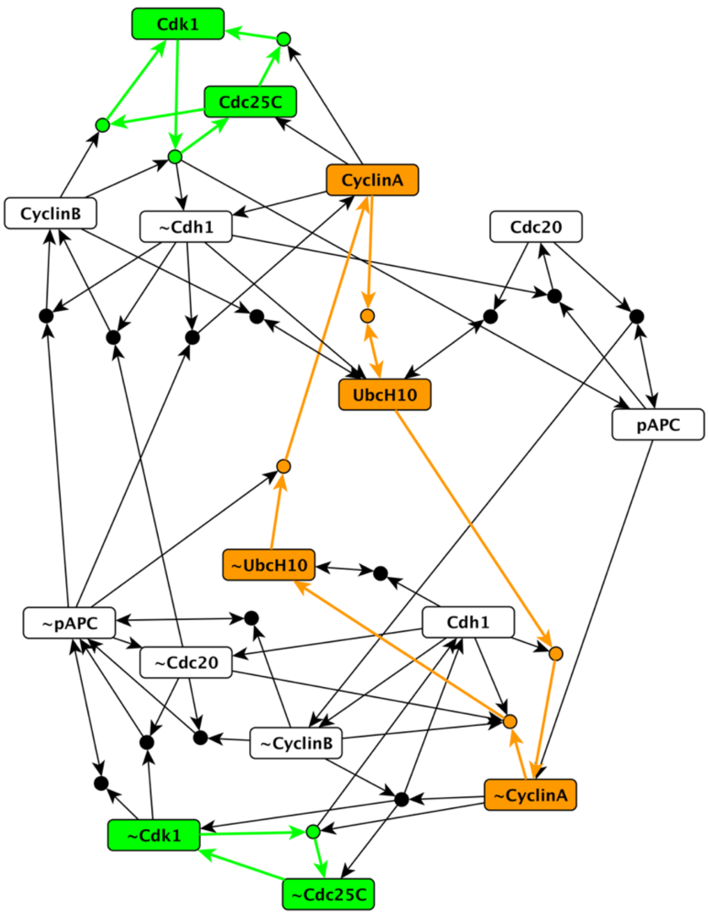

Supplementary Figure S6.1. The expanded network of the Phase Switch Oscillator embodies the logic relationships that drive its oscillating behavior. Colored nodes and edges highlight two characteristic subgraphs of the expanded network. The green nodes and edges indicate the subgraphs corresponding to the positive feedback loop between Cdk1 and Cdc25C (bidirectional edge in Figure 4 bottom panel). There are two overlapping cycles (i.e. closed paths with non-repeating virtual or composite nodes), both of length four. Each cycle involves Cdk1, Cdc25C and two composite nodes (one shared by both cycles). These cycles indicate that the positive feedback can only sustain the on (1) state of Cdk1 and Cdc25C if CyclinB (for one of the cycles) or both CyclinA and CyclinB (for the other cycle) are also simultaneously on. There is a consistent cycle formed by ~Cdk1, ~Cdc25C and a composite node that receives input from ~CyclinA; this means that Cdk1 and Cdc25 can simultaneously sustain their off (0) state if CyclinA is also off. The orange nodes and edges highlight the subgraph that corresponds to the negative feedback loop (bidirectional edge) between CyclinA and UbcH10 (Figure 4 bottom panel). This subgraph is a cycle of length eight; it contains both virtual nodes of CyclinA and UbcH10 as well as four composite nodes. In general, negative feedback loops result in a cycle in the expanded network involving both states of the involved nodes; we call this type of cycle inconsistent cycle. Positive feedbacks form two disjoint (groups of) cycles. Each of these cycles is consistent. The disjoint cycles have opposite states and can have different conditions.

The average in-degree of the expanded network is less than two, markedly smaller than the average in-degree of the original Phase Switch Oscillator network, which is 3.5. This illustrates that regulators need to cooperate to induce state changes in target nodes [S36, S37]. The whole expanded network is an oscillating motif: it is strongly connected, it is composite-closed, it contains the complementary of each virtual node, and it does not have any stable motifs. As shown on Supplementary Figure S6.2, the expanded network has more than 6000 cycles (closed paths with non-repeating virtual or composite nodes), the vast majority of which are inconsistent (i.e. they contain an internal contradiction either in the virtual or composite nodes of the cycle; see orange nodes and edges in Supplementary Figure S6.1). There are 28 consistent cycles

(similar to the green cycle in Supplementary Figure S6.1), all of which have fewer than 8 nodes. All the inconsistent cycles have 8 or more nodes. This difference in cycle sizes indicates that more conditions (in terms of the state of other nodes) need to be satisfied to ensure the oscillation of a node than a sustained state of a node in the Phase Switch Oscillator. In other words, nodes must rely on each other to achieve a sustained oscillation. Supplementary Note S7 indicates examples of minimal subgraphs through which a virtual node (in combination with other virtual nodes) can induce its own negation.

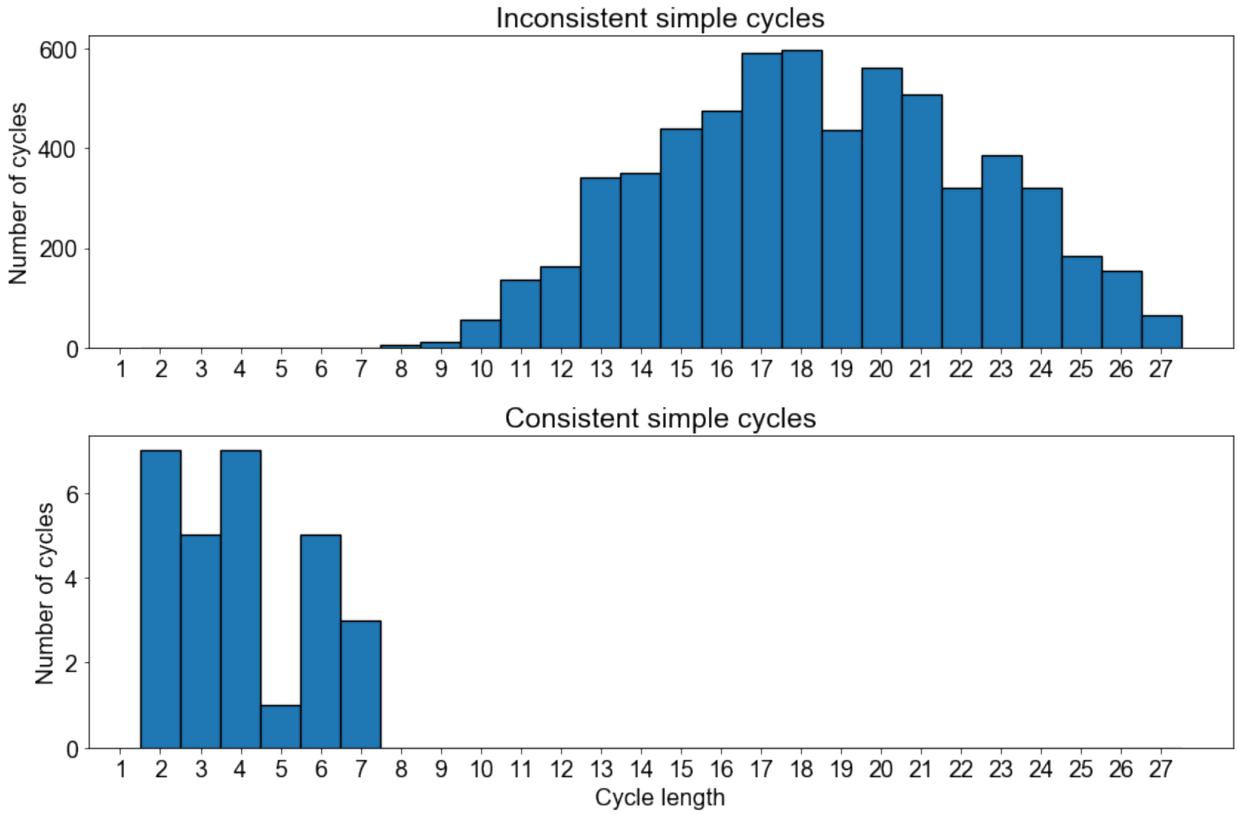

Supplementary Figure S6.2. Distribution of the length of consistent (bottom) and inconsistent (top) cycles on the expanded network. As illustrated by the green nodes and edges in Supplementary Figure S6.1, consistent cycles correspond to positive feedback loops in the regulatory network. They are the building blocks of conditionally stable motifs. We define inconsistency as the involvement of two opposite states of the same node, either as virtual nodes or as determinants of a composite node. Inconsistent cycles are akin to traversing a negative feedback loop of the regulatory network twice (as illustrated by the orange nodes and edges in Supplementary Figure S6.1).

To help identify nodes that play a key role in the complex attractor, we use the information in the expanded network to construct a weighted network of virtual nodes in which the edge weight between two virtual nodes depends on the probability that one is sufficient for the other. If a virtual node *u* does not regulate the virtual node *v* no edge is placed. Otherwise, we calculate the sufficiency probability *s(u, v)* that u is sufficient for v, assuming that all other nodes take each of their two values with 50% probability. We

assign an edge weight of 1/*s(u, v)* for each ordered pair (*u,v*). Intuitively, the higher the sufficiency of an edge, the closer the parent node is to the child node, which is represented as a lower edge weight. As a concrete example, let *u* be Cdk1=1 and *v* be pAPC=1. The regulatory function for pAPC is

f_{pAPC}= CyclinB and Cdk1 or pAPC and Cdc20.

Cdk1=1 is sufficient for pAPC=1 if and only if CyclinB is active, which we assume occurs with 50% probability. Therefore, *s(u, v)* is 0.5, and so we assign a weight of 2 to the edge from Cdk1=1 to pAPC=1.

After this network is constructed, we compute the betweenness centrality score for each virtual node. The betweenness centrality of a node, *i*, is the sum over all node pairs (*j*,*k*) of the fraction of shortest paths from *j* to *k* that pass through *i* [S38]. As frequently done, we divide this score by the highest observed score, such that the values are between 0 and 1. The relative scores estimate the likelihood that the associated node state will be achieved as information spreads through the network from one randomly selected node to another. We view this as a proxy for the importance of a node in sustaining the observed oscillation.

Supplementary Figure S6.3 shows the weighted network of virtual nodes, wherein the relative betweenness centrality of each node is indicated next to each virtual node. Both virtual nodes of CyclinA, pAPC, and Cdh1 have a near-maximal betweenness centrality. At the other extreme, the virtual nodes Cdc25C, ~Cdc25C and ~UbcH10 have a betweenness centrality of 0.

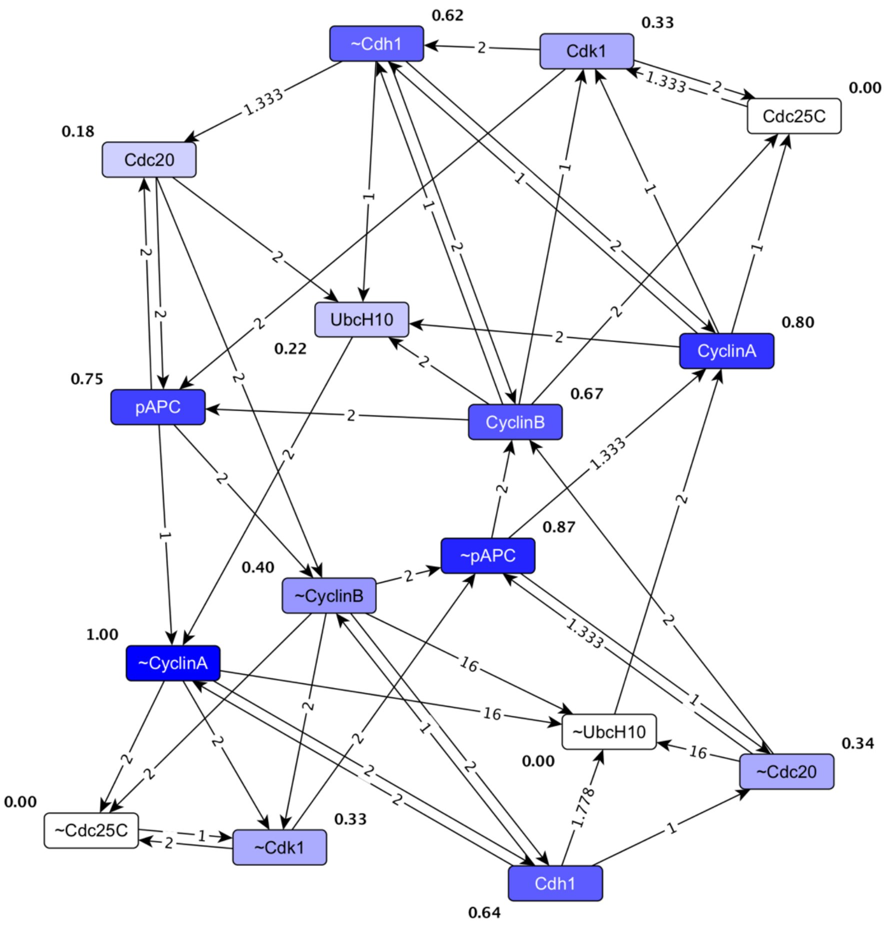

Supplementary Figure S6.3. The virtual nodes of the expanded network contribute unequally to the oscillating motif. The weights of the edges among virtual nodes indicate the inverse of the probability of sufficiency. The edges with high sufficiency (weight below 2) correspond to pairs of virtual nodes that are connected directly or through multiple

composite nodes. Virtual nodes marked by the node names preceded with ~ indicate the OFF state of the corresponding node. Node color represents the betweenness centrality, which is also indicated numerically beside each node. A darker shade indicates a higher betweenness centrality.

Supplementary Note S7. Conditionally stable motifs of the Phase Switch Oscillator can cause the destabilization of their own conditions

We determine the logic implications of the stabilization of the states of a conditionally stable motif (CSM) using the concept of the logic domain of influence (LDOI). The LDOI of a given "seed" set of virtual nodes is the set of node states that are causally stabilized by the seed set when it is held fixed [66]. We used the LDOI identification algorithm developed by Yang et al., available at https://github.com/yanggangthu/BooleanDOI. In this algorithm the LDOI is defined and built via an iterative process on the expanded network, beginning with the empty set. On each iteration, every child node of every virtual node in the seed set is considered (breadth first) and added to the LDOI set if the child node does not contradict any nodes in the seed set and either 1) the child node is a virtual node or 2) the child node is a composite node and all its parent nodes are already in the set. This process is continued, considering child nodes of the nodes already included in the LDOI, until no new nodes can be added. The LDOI of a stable motif includes the motif itself and no contradictions.

We found that the LDOI of five CSMs includes the complementary of the virtual node that serves as the condition of the CSM. This means that the CSM sooner or later leads to a contradiction with its own condition, thus to its own deactivation. These five CSMs are indicated in Supplementary Table S7.1. In addition, Supplementary Figure S7.1 illustrates the LDOI of CSM 3 on the expanded network.

Supplementary Table S7.1. The conditionally CSMs of the PSO network that cause their own destabilization. The first column is the identifier of the CSM. The second column contains the node states (virtual nodes on the expanded network where ~ refers to the 0 state) making up the CSM. The third column is the state that serves as the condition that needs to be sustained for the CSM to be stable. The opposite state is contained in the logic domain of influence of the CSM. Such destabilization does not happen in the Phase Switch because the stable motifs either stabilize, or violate, the condition of each CSM (see Figure 3).

Name	Virtual nodes of the conditionally stable motif	Virtual node that serves as condition
C3	~CyclinA, Cdh1, ~CyclinB	UbcH10
C4	~CyclinA, ~Cdk1, Cdh1, ~CyclinB	UbcH10
C5	~CyclinA, ~Cdk1, Cdh1, ~Cdc25C	UbcH10
C11	~Cdh1, CyclinB, Cdk1, Cdc25C	~Cdc20 or ~pAPC
C13	pAPC	Cdc20
C14	pAPC, Cdc20	~Cdh1

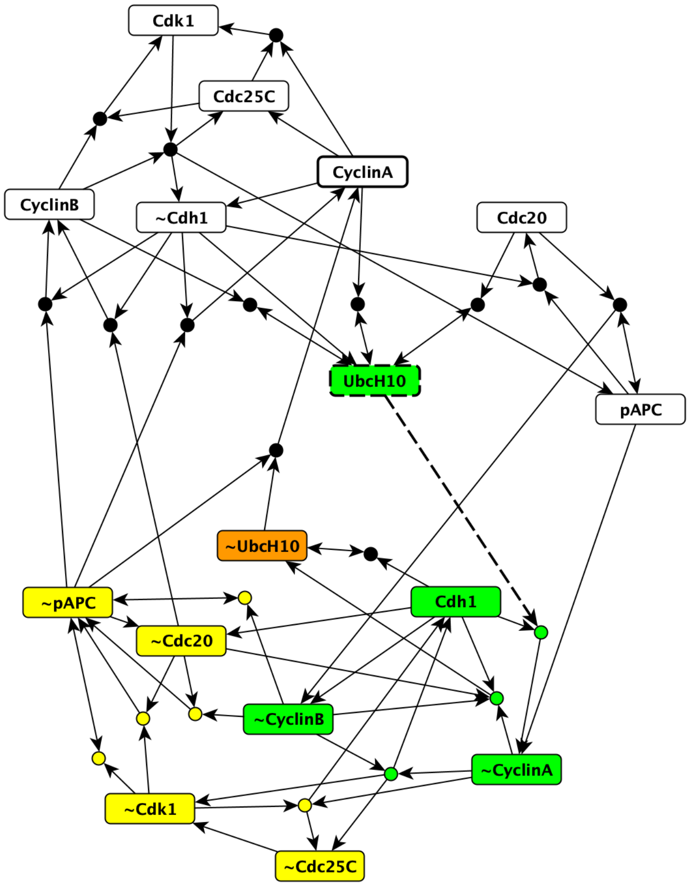

Supplementary Figure S7.2. Illustration of a conditionally stable motif causing its own destabilization. The conditionally stable motif C3, highlighted in green, is conditioned on UbcH10, emphasized with the dashed outline. The yellow nodes are the logical domain of influence (LDOI) of C3, i.e. states that will stabilize as long as C3 is stable. The on state of ~UbcH10, highlighted with orange, is also part of the LDOI but marks the destabilization of C3.

Supplementary Note S8. Detailed description of the agreement of the expanded network and complex attractor of the Phase Switch Oscillator

To illustrate the correspondence between the expanded network (Supplementary Figures S6.1, S7.2) and the complex attractor (Figure 5) of the Phase Switch Oscillator, we follow the complex attractor, starting with the G0/G1 state. As can be seen from Supplementary Table S2, in this state the ~Cyc, ~Cyclosome and ~UbcH10 conditionally stable motifs (CSMs) are active. As the latter two CSMs represent the violation of the conditions of ~Cyc (see Figure 8), it will destabilize with certainty. Specifically, the expanded network indicates that the condition for CyclinA to turn ON (i.e. the condition for the virtual node CyclinA to be reached) is that UbcH10 and pAPC are simultaneously off (this is expressed by a composite node whose regulators are ~UbcH10 and ~pAPC) or Cdc20 and Cdh1 are simultaneously off. The first of these conditions is satisfied in the G0/G1 state, thus CyclinA will turn ON with certainty, in agreement with and explaining the probability 1 of the edge between the G0/G1 state and the state with overlap triple (7,4,1). Cdc25C turns on and Cdh1 turns off in arbitrary order between the state with overlap (7,4,1) and the state (5,6,3) because Cdc25C and

~Cdh1 are driven by CyclinA (i.e. there is an edge from CyclinA to Cdc25C and an edge from CyclinA to ~Cdh1). UbcH10 is driven by ~Cdh1, thus its state transition follows that of Cdh1. Thus the ~UbcH10 CSM has been replaced with the UbcH10 CSM. Cdk1 turns on if CyclinA and Cdc25C are simultaneously on, thus its state transition follows the turning on of Cdc25C. The turning ON of CyclinB needs the simultaneous OFF state of Cdh1 and pAPC or of Cdh1 and Cdc20 (indicated by two separate composite nodes). Both pAPC and Cdc20 are off in the state (7,4,1), thus the state change of CyclinB follows that of Cdh1, taking the system to the post-G2 state. In this state the Cyc CSM has completely established, and will stay active as long as ~Cyclosome is active. The multiple orders in which the previously described five nodes can change state induces the possibility of paths from the state (7,4,1) to the post-G2 state that skip the state (5,6,3).

The turning ON of pAPC necessitates CyclinB and Cdk1 (see composite node), both of which are present in the post-G2 state, thus this will happen with high probability. The expanded network's subgraph that starts with ~pAPC and CyclinA, contains Cdc25C, ~Cdh1, Cdk1, CyclinB as well as 3 composite nodes, and converges on pAPC, expresses the minimal logical condition for the mediated self-inhibition of pAPC. This subgraph is subsumed in the path from ~Cylosome to Cyc and then to Cyclosome in Figure 9. The combination of pAPC and ~Cdh1 leads to the turning ON of Cdc20. Cdc20 contributes to the self-sustained expression of pAPC; thus the Cyclosome CSM is now established. This destabilizes the Cyc CSM, starting with the turning OFF of CyclinA driven by pAPC. The trajectory of the system has now converged into the post-SAC state. The subgraph of the expanded network that starts with CyclinA and CyclinB and contains Cdc25C, Cdk1, pAPC and three composite nodes, ends in ~CyclinA. This subgraph contributes to the path from Cyc to Cyclosome and then to ~Cyc in Figure 9.

CyclinB turns off if Cdc20 and pAPC are simultaneously present. The subgraph that starts with CyclinB and Cdk1 and contains ~Cdh1, pAPC (both of which are regulated by the same composite node), Cdc20 and two additional composite nodes, and ends in ~CyclinB, expresses the mediated self-inhibition of CyclinB. This subgraph is another contributor to the path from Cyc to Cyclosome and then to ~Cyc. The virtual nodes ~CyclinA and ~CyclinB together regulate a composite node that drives all three of Cdh1, ~Cdk1 and ~Cdc25C. This is the reason these three nodes switch states in arbitrary order between the state marked (2,3,6) and (5,2,3), while with synchronous update the second state is a direct successor of the first. In the state with overlap triple (5,2,3) the ~Cyc CSM has established. Cdh1 is sufficient to turn off Cdc20; this in certain trajectories can happen before the turning off of Cdc25C or Cdk1, contributing to the possibility of a trajectory from state (2,3,6) directly to (6,3,4). The OFF state of Cdc20, combined with ~CyclinB or ~Cdk1, drive ~pAPC. At this point the ~Cyclosome CSM has established. The turning off of UbcH10 necessitates the combination of ~CyclinA, ~CyclinB, Cdh1, ~Cdc20. Since all of these node states were reached in the previous steps, the turning off of pAPC and UbcH10 can happen in arbitrary order. The system has now returned to the G0/G1 state.

This step-by-step comparison helps us see that the reduced degree of freedom of the complex attractor in terms of what node changes state next is because of the strong inter-dependence of nodes. Nodes within Cyc and Cyclosome have a strong

positive inter-dependence. Cyc forms negative feedback loops with Cyclosome and UbcH10, which yields a negative inter-dependence.

Supplementary Note S9. Analyzing the effect of locking a node state on the attractor repertoire of the Phase Switch Oscillator

To evaluate each node state's contribution to the complex attractor, we systematically set each node in its active or inactive state and identify the motif structure and attractor repertoire of the thus-modified dynamical system. The modified systems' dynamic behaviors fall into three categories: 1) the PSO oscillation is preserved as the sole attractor, 2) the modified system has a single point attractor, or 3) the modified system has multiple point attractors. The expanded networks for representatives of each of the three categories are shown in Figure 10, alongside the original system's expanded network. A comprehensive summary of the stable and conditional motifs and attractors corresponding to each intervention is provided in Supplementary Table S4. Of the sixteen possible node-state-fixing interventions, Cdc25c = 1, UbcH10 = 0, and Cdk1 = 1 preserve oscillation. A shared feature of these three interventions is that they are not conditions for any CSM and cannot stabilize any other node. Therefore, no stable motif is created by any of these interventions. For example, the system modified by locking in UbcH10=0 loses the CSMs that include UbcH10 as a virtual node or as a condition (that is, it loses motifs C4, C5 and C12). Nevertheless, sufficiently many CSMs that induce their own destabilization remain so that the oscillation is preserved.

Eleven of the node-fixing interventions result in a reduced system that has a single point attractor (i.e., it is monostable). Most newly stabilized states are highly similar to the three Phase Switch Attractors. Namely, CyclinA = 0, pAPC = 0 and UbcH10=1 each lock the PSO into a G0/G1-like state; Cdc25c = 0, Cdk1 = 0 and CyclinB = 0 each lock it into a G2-like attractor, while Cdc20 = 0 locks it into SAC (see Supplementary Table S4). In contrast, Cdh1 = 1 stabilizes the network in a state poised to transition out of G1 into G2, CyclinA = 1 locks it poised at the boundary of G2 and mitosis (towards SAC), and Cdh1 = 0 locks it at the boundary between SAC and G0/G1. In all the cases of monostability a CSM or the union of multiple compatible CSMs becomes a stable motif, thus its influence becomes non-contradictory; and opposing CSMs disappear. In addition, two modifications result in multi-stability; Cdc20 = 1 and CyclinB = 1. Locking Cdc20 to 1 results in three qualitatively similar point attractors that only differ in the composition of the protein complex containing Cdc20 (i.e., they only differ in the values of pAPC and UbcH10); the rest of the nodes stabilize into their G0/G1 state.

In contrast, locking CyclinB on creates two highly dissimilar point attractors that differ in five node states and overlap only in UbcH10 = 1 and CyclinA = 0 (see Figure 10). The bistability in the presence of forced CyclinB expression is due to the fact that sustained CyclinB is the (direct or indirect) condition for two mutually exclusive CSMs. CyclinB is the condition for the C10 CSM (Cdk1, Cdc25C), which drives an attractor resembling a cell at the point of SAC passage (brown node-states on Figure 10, top right). CyclinB is also the condition of the C12 CSM (UbcH10), which when stabilized serves as the condition for the C3 CSM (Cdh1, ~CyclinA, ~Cdc25C, ~Cdk1), which when stabilized leads to an attractor resembling the G0/G1 state (blue node-states on

Figure 10, top right). In contrast, when CyclinB is held inactive the C7 CSM (~Cdc20, pAPC) becomes a stable motif. The activation of this motif enables the activation of the C9 CSM (CyclinA, ~Cdh1), resulting in a single point attractor most similar to the G2 attractor of the Phase Switch.

References

43. Yao G, Lee TJ, Mori S, Nevins JR, You L. A bistable Rb-E2F switch underlies the restriction point. Nat Cell Biol. 2008;10: 476–482.

S1. Yao G, Tan C, West M, Nevins JR, You L. Origin of bistability underlying mammalian cell cycle entry. Mol Syst Biol. 2011;7: 485.

S2. Alfieri R, Barberis M, Chiaradonna F, Gaglio D, Milanesi L, Vanoni M, et al. Towards a systems biology approach to mammalian cell cycle: modeling the entrance into S phase of quiescent fibroblasts after serum stimulation. BMC Bioinformatics. 2009;10 Suppl 12: S16.

S3. Pfeuty B. Strategic cell-cycle regulatory features that provide mammalian cells with tunable G1 length and reversible G1 arrest. PLoS One. 2012;7: e35291.

S4. Giunta S, Jackson SP. Give me a break, but not in mitosis. Cell Cycle. 2011. pp. 1215–1221. doi:10.4161/cc.10.8.15334

S5. Pardee AB. A restriction point for control of normal animal cell proliferation. Proc Natl Acad Sci U S A. 1974;71: 1286–1290.

S6. Zoncu R, Efeyan A, Sabatini DM. mTOR: from growth signal integration to cancer, diabetes and ageing. Nat Rev Mol Cell Biol. 2011;12: 21–35.

S7. Varsano G, Wang Y, Wu M. Probing Mammalian Cell Size Homeostasis by Channel-Assisted Cell Reshaping. Cell Rep. 2017;20: 397–410.

S8. Shaltiel IA, Krenning L, Bruinsma W, Medema RH. The same, only different - DNA damage checkpoints and their reversal throughout the cell cycle. J Cell Sci. 2015;128: 607–620.

19. Sizek H, Hamel A, Deritei D, Campbell S, Regan ER. Boolean model of growth signaling, cell cycle and apoptosis predicts the molecular mechanism of aberrant cell cycle progression driven by hyperactive PI3K. PLOS Computational Biology. 2019; 15: e1006402.

S9. Hsieh H-J, Zhang W, Lin S-H, Yang W-H, Wang J-Z, Shen J, et al. Systems biology approach reveals a link between mTORC1 and G2/M DNA damage checkpoint recovery. Nat Commun. 2018;9: 3982.

S10. Manchado E, Eguren M, Malumbres M. The anaphase-promoting complex/cyclosome (APC/C): cell-cycle-dependent and -independent functions. Biochem Soc Trans. 2010;38: 65–71.

S11. Peters J-M. The anaphase promoting complex/cyclosome: a machine designed to destroy. Nat Rev Mol Cell Biol. 2006;7: 644–656.

S12. Qiao X, Zhang L, Gamper AM, Fujita T, Wan Y. APC/C-Cdh1: from cell cycle to cellular differentiation and genomic integrity. Cell Cycle. 2010;9: 3904–3912.

S13. Reddy SK, Rape M, Margansky WA, Kirschner MW. Ubiquitination by the

anaphase-promoting complex drives spindle checkpoint inactivation. Nature. 2007;446: 921–925.
S14. Nezi L, Musacchio A. Sister chromatid tension and the spindle assembly checkpoint. Current Opinion in Cell Biology. 2009. pp. 785–795.
S15. Thornton BR, Toczyski DP. Precise destruction: an emerging picture of the APC. Genes Dev. 2006;20: 3069–3078.
S16. Donzelli M, Squatrito M, Ganoth D, Hershko A, Pagano M, Draetta GF. Dual mode of degradation of Cdc25 A phosphatase. EMBO J. 2002;21: 4875–4884.
S17. Hoffmann I, Draetta G, Karsenti E. Activation of the phosphatase activity of human Cdc25A by a Cdk2-Cyclin E dependent phosphorylation at the G1/S transition. The EMBO Journal. 1994;13; 4302–4310.
S18. Frazer C, Young PG. Phosphorylation Mediated Regulation of Cdc25 Activity, Localization and Stability. In: Protein Phosphorylation in Human Health. 2012.
S19. Karaïskou A, Cayla X, Haccard O, Jessus C, Ozon R. MPF amplification in Xenopus oocyte extracts depends on a two-step activation of Cdc25 phosphatase. Exp Cell Res. 1998;244: 491–500.
S20. Hoffmann I, Clarke PR, Marcote MJ, Karsenti E, Draetta G. Phosphorylation and activation of human Cdc25-C by Cdc2--Cyclin B and its involvement in the self-amplification of MPF at mitosis. EMBO J. 1993;12: 53–63.
S21. Harper JW. The anaphase-promoting complex: it's not just for mitosis any more. Genes & Development. 2002. pp. 2179–2206.
S22. Jackman MR, Pines JN. Cyclins and the G2/M transition. Cancer Surv. 1997;29: 47–73.
S23. Heald R, McLoughlin M, McKeon F. Human wee1 maintains mitotic timing by protecting the nucleus from cytoplasmically activated Cdc2 kinase. Cell. 1993;74: 463–474.
S24. Deibler RW, Kirschner MW. Quantitative reconstitution of mitotic CDK1 activation in somatic cell extracts. Mol Cell. 2010;37: 753–767.
S25. Blomberg I, Hoffmann I. Ectopic Expression of Cdc25A Accelerates the G1/S Transition and Leads to Premature Activation of Cyclin E- and Cyclin A-Dependent Kinases. Molecular and Cellular Biology. 1999. pp. 6183–6194.
S26. Katsuno Y, Suzuki A, Sugimura K, Okumura K, Zineldeen DH, Shimada M, et al. Cyclin A-Cdk1 regulates the origin firing program in mammalian cells. Proc Natl Acad Sci U S A. 2009;106: 3184–3189.
S27. Yam CH, Fung TK, Poon RYC. Cyclin A in cell cycle control and cancer. Cell Mol Life Sci. 2002;59: 1317–1326.
12. Fauré A, Naldi A, Chaouiya C, Thieffry D. Dynamical analysis of a generic Boolean model for the control of the mammalian cell cycle. Bioinformatics. 2006;22: e124–31.
S28. Rape M, Kirschner MW. Autonomous regulation of the anaphase-promoting complex couples mitosis to S-phase entry. Nature. 2004;432: 588–595.
S29. He E, Kapuy O, Oliveira RA, Uhlmann F, Tyson JJ, Novák B. System-level feedbacks make the anaphase switch irreversible. Proc Natl Acad Sci U S A.

2011;108: 10016–10021.

S30. Chen RH, Waters JC, Salmon ED, Murray AW. Association of spindle assembly checkpoint component XMAD2 with unattached kinetochores. Science. 1996;274: 242–246.

S31. Musacchio A, Salmon ED. The spindle-assembly checkpoint in space and time. Nat Rev Mol Cell Biol. 2007;8: 379–393.

S32. Rudner AD, Murray AW. Phosphorylation by Cdc28 Activates the Cdc20-Dependent Activity of the Anaphase-Promoting Complex. The Journal of Cell Biology. 2000; 1377–1390.

S33. Chen X & Prywes R Serum-induced expression of the *Cdc25a* gene by relief of e2f-mediated repression. Molecular and cellular biology 1999;19:4695–4702.

S34. Helin K Regulation of cell proliferation by the E2f transcription factors. Current opinion in genetics & development 1998; 8:28–35.

S35. Lee J, Kumagai A & Dunphy WG Positive regulation of Wee1 by Chk1 and 14-3-3 proteins. Molecular biology of the cell 2001; 12:551–563.

17. Deritei D, Aird, WC, Ercsey-Ravasz M & Regan, ER Principles of dynamical modularity in biological regulatory networks. Scientific Reports 2016;6: 21957.

S36. Marques-Pita M & Rocha L M. Canalization and control in automata networks: body segmentation in Drosophila melanogaster. PLoS One 2013; 8:e55946.

S37. Li Y, Adeyeye J O, Murrugarra D., Aguilar B. & Laubenbacher R. Boolean nested canalizing functions: A comprehensive analysis. Theoretical Computer Science 2013; 481:24–36.

S38. Freeman L C. A Set of Measures of Centrality Based on Betweenness. Sociometry 40, 35 (1977).

66. Yang G, Zañudo J. G. T. & Albert, R. Target Control in Logical Models Using the Domain of Influence of Nodes. Frontiers in Physiology 9, (2018).

S39. Garg A, Di Cara A, Xenarios I, Mendoza L, and De Micheli G. Synchronous versus asynchronous modeling of gene regulatory networks. Bioinformatics 24, no. 17 (2008): 1917-1925.

S40. Greil F and Drossel B. Dynamics of critical Kauffman networks under asynchronous stochastic update. Physical Review Letters 95, no. 4 (2005): 048701.

S41. Saadatpour A, Albert I and Albert R. Attractor analysis of asynchronous Boolean models of signal transduction networks. Journal of theoretical biology 266, no. 4 (2010): 641-656.

S42. Li S, Assmann SM, Albert R. Predicting essential components of signal transduction networks: a dynamic model of guard cell abscisic acid signaling. PLoS Biology 4, e312 (2006).
