# Supplementary material for: A feedback loop of conditionally stable circuits drives the cell cycle from checkpoint to checkpoint
Source: Sci Rep. 2019 Nov 11;9:16430. doi: 10.1038/s41598-019-52725-1 (PMC6848090; doi:10.1038/s41598-019-52725-1)
Supplement: Supplementary file 4 — Supplementary Note S10 [file 41598_2019_52725_MOESM4_ESM.pdf]

Glossary of
A feedback loop of conditionally stable circuits drives the cell cycle from checkpoint to checkpoint

Dávid Deritei[1,2], Jordan Rozum[1], Erzsébet Ravasz Regan[3], Réka Albert[1]

[1]Department of Physics, Pennsylvania State University, University Park, PA, United States of America
[2] Department of Network and Data Science, Central European University, Budapest, Hungary
[3] Biochemistry and Molecular Biology, The College of Wooster, Wooster, OH, United States of America

Boolean Dynamics

synchronous update scheme: all nodes are updated at the same time and their next state is determined by the previous state of the system

general asynchronous update scheme: the next node to be updated is chosen randomly

attractor: a single state, or a set of states that the system keeps revisiting indefinitely

complex attractor: an attractor with multiple states in general asynchronous update

limit cycle: an attractor with multiple states in synchronous update

state transition graph (STG): the network representing all the possible transitions between the states of a system

Network Theory

strongly connected graph: a graph for which there is a path between every pair of nodes

betweenness centrality: the betweenness centrality of a node, v, is the fraction of shortest paths (between all pairs of nodes that do not include v) that pass through v

Expanded Network

expanded network: encodes the causal relationships between node states reflected in the regulatory functions. It consists of two "virtual nodes" for each node (one for each of the two possible states) and "composite nodes" that embody AND gates among two or more node states. An edge from a virtual node to a composite node indicates that the virtual node is a necessary condition for states described by the composite node. An edge from any node to a virtual node indicates that the parent node is a sufficient condition for the state represented by the child node.

virtual node: a node in an expanded network that represents either the "ON" or "OFF" state of a single variable

composite node: a node in an expanded network that represents an AND gate

consistent subgraph of the expanded network: a subgraph for which all represented conditions can be simultaneously satisfied

composite-closed subgraph of the expanded network: a subgraph with the property that if a composite node is in the subgraph, so too are all its virtual node parents

stable motif: a subgraph of an expanded network that satisfies four properties: 1) it is strongly connected, 2) it is consistent, 3) it is composite-closed, and 4) it is minima*l* (it contains no subgraphs, other than itself, satisfying the first three properties)

oscillating motif: a subgraph of an expanded network that satisfies five properties: 1) it is strongly connected, 2) it is composite-closed*,* 3) each of its virtual nodes is contradicted by another one of its virtual nodes, 4) it contains no stable motifs, and 5) it is minimal (it contains no nontrivial subgraphs satisfying the first four properties)

logic domain of influence (LDOI): the LDOI of a seed set of virtual nodes is the set of node states that are causally stabilized by the seed set when it is held fixed

Conditionally Stable Motifs

conditionally stable motif (CSM): a strongly connected subgraph of the expanded network that, together with the parents of all its composite nodes, is consistent

CSM states: the virtual node states contained within a CSM

CSM conditions: the parents of the composite nodes in the CSM that are not themselves members of the CSM. Note: if the conditions of a CSM are held fixed, the CSM becomes a stable motif.

cycle graph: a graph constructed to help identify CSMs; each node represents a consistent cycle in the expanded network, and edges are drawn between the nodes of the cycle graph if the corresponding cycles in the expanded network are mutually consistent and share a node

Biological Definitions

G0: cell state corresponding to quiescent cells

G1: first growth phase of the cell cycle. In the Phase Switch, a cell arrested in the G0 or G1 phase is reflected by a point attractor where every node except Cdh1 is inactive. The Phase Switch oscillator also passes through a similar state.

restriction point: checkpoint to verify that the cells are ready to begin DNA synthesis

G2: second growth phase of the cell cycle, before clearing the DNA damage checkpoint. In the Phase Switch, a cell arrested in this phase is reflected by a point attractor in which CyclinA, CyclinB and Wee1 are active. The Phase Switch Oscillator passes through a post-G2 state in which CyclinA, CyclinB and Cdk1 are active.

mitosis: the process when the chromosomes separate and are pulled apart by microtubules.

spindle assembly checkpoint (SAC): the checkpoint to verify that the spindle that will pull sister chromatids apart is assembled. The Phase Switch represents cells arrested before clearing the SAC by a point attractor in which CyclinB, Mad2 and pAPC are active and CyclinA is inactive. The Phase Switch Oscillator passes through a near-SAC state (where CyclinA is still active).

Miscellaneous

state overlap: for two states X and Y of a system with N nodes, the overlap is the number of node states that are in agreement; this is equal to N - H(X,Y), where H is the Hamming distance

overlap triple: for a given state X, the overlap triple is an ordered triple that contains the state overlap of X with the reference states G0/G1, G2, and SAC, in that order
